# Uncovering Structural Opportunities for Zirconium Metal–Organic Frameworks via Linker Desymmetrization

**DOI:** 10.1002/advs.201901855

**Published:** 2019-09-30

**Authors:** Yutong Wang, Liang Feng, Kai Zhang, Kun‐Yu Wang, Weidong Fan, Xiaokang Wang, Bingbing Guo, Fangna Dai, Liangliang Zhang, Daofeng Sun, Hong‐Cai Zhou

**Affiliations:** ^1^ College of Science School of Materials Science and Engineering China University of Petroleum (East China) Qingdao Shandong 266580 China; ^2^ Department of Chemistry Texas A&M University College Station TX 77843 USA; ^3^ Xi'an Institute of Flexible Electronics Northwestern Polytechnical University Xi'an 710072 China; ^4^ Department of Materials Science and Engineering Texas A&M University College Station TX 77843‐3003 USA

**Keywords:** linker design, linker desymmetrization, metal–organic frameworks, topology, zirconium

## Abstract

The discovery of metal–organic frameworks (MOFs) mimicking inorganic minerals with intricate topologies requires elaborate linker design guidelines. Herein, the concept of linker desymmetrization into the design of tetratopic linker based Zr‐MOFs is applied. A series of bent tetratopic linkers with various substituents are utilized to construct Zr‐MOFs with distinct cluster connectivities and topologies. For example, the assembly between a bent linker L‐SO_2_ with *C*_2v_ symmetry and an 8‐connected Zr_6_ cluster leads to the formation of an **scu** topology, while another **flu** topology can be obtained by the combination of a novel 8‐connected Zr_6_ cluster and a bent linker L‐O with *C*_1_ symmetry. Further utilization of restricted bent linker [(L‐(CH_3_)_6_)] gives rise to a fascinating (4, 6)‐c **cor** net, originated from the corundum lattice, with an unprecedented 6‐c Zr_6_ cluster. In addition, the removal of toxic selenite ions in aqueous solution is performed by PCN‐903‐(CH_3_)_6_ which exhibits rapid and efficient detoxification. This work uncovers new structural opportunities for Zr‐MOFs via linker desymmetrization and provides novel design strategies for the discovery of sophisticated topologies for practical applications.

## Graphical abstract

A series of bent tetratopic linkers with various substituents are utilized to construct zirconium metal–organic frameworks (Zr‐MOFs) with distinct cluster connectivities and topologies including **scu, flu,** and **cor** topology. The discovery here uncovers new structural opportunities for Zr‐MOFs via linker desymmetrization and provides novel design strategies for the discovery of sophisticated topologies for practical applications.

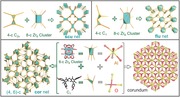

Metal−organic frameworks (MOFs), or porous coordination polymers, are a well‐developed class of porous crystalline materials constructed from inorganic metal clusters and organic linkers.^1^, ^2^, ^3^, ^4^, ^5^, ^6^, ^7^, ^8^, ^9^ As highly tunable structures through judicious linker design, MOFs attracted considerable interests in fields, such as gas storage, separation, heterogeneous catalysis, sensing, light harvesting, and drug delivery.^4^, ^10^, ^11^ Among the numerous MOFs, zirconium MOFs have received increasing attention in recent years due to their highly stable feature originated from the robust Zr—O cooridnation bonds.^1^, ^12^ The high chemical stability of Zr‐MOF ensures its economical large‐scale production in water solution and also permits its structural intactness when exposed to harsh conditions.

The combination of Zr clusters and different organic linkers has contributed to the structural diversity and functional complexity of the MOF materials. Various synthetic approaches have been developed to synthesize Zr‐MOFs with structural complexicity, including linker design strategies, pillar strategies, postsynthetic methods, and preformed clusters.^1^ For example, Zhou group reported the steric control over ditopic linker based Zr‐PCN‐700, where bulky substituents on the 2‐ and 2′‐positions of BPDC (biphenyl‐4,4′‐dicarboxylate) were introduced to constrain the two carboxylates and phenyl rings into a perpendicular conformation.^13^ Later Guillerm and co‐workers descibed the topoligical influence of zigzag ligands during the assembly of **bcu** net.^14^ Speaking of 4‐connected linkers, the structural diversity based on Zr_6_ clusters can also be expanded by tuning linker conformation. For example, various topologies including **ftw**, **csq**, **shp**, **scu**, **she**, and **sqc** can be accessed when square or rectangle tetratopic linkers are chosen.^15^, ^16^, ^17^, ^18^, ^19^, ^20^ The effects of linker geometry and flexibility influenced by the bulkiness of substituents can also be utilized to enhance the diversity of Zr‐tetratopic carboxylate MOFs.^21^ However, how to continue enhancing the structural and compositional compilation of Zr‐tetratopic carboxylate MOFs poses a synthetic challenge.

Herein, we initiate a systematic study on Zr‐tetracarboxylate frameworks constructed from a series of bent tetratopic linkers with various substituents. The utilization of a bent ligand with lower symmetry overcomes the traditional topology problems limited by the rigid ligand backbones. The tunable linker geometries and conformations here by altering the composition of substituents generate a series of Zr‐MOFs with varying topologies including **scu**, **flu,** and **cor**. This work provides fresh insights into the discovery of Zr‐tetracarboxylate frameworks with unprecedented topologies and conformations of building blocks, and highlights the importance of linker desymmetrization for dictating MOF architectures.

As shown in **Figure** **1**a, four bent tetratopic ligands with various substituents (R_1_ = —H, —CH_3_; R_2_ = —NH_2_, —CH_3_; R_3_ = —SO_2_, —CH_2_, —O) were selected to construct Zr‐based MOFs. Due to the bulkiness differences of R_1_ substituents, the distances and angles between two central rings can be tuned. The presence of R_2_ substituents can effectively influence the rotation angles between two peripheral phenyl rings near the central ring, while R_3_ substituents are utilized to tune rotation degrees around the linker center.

**Figure 1:**
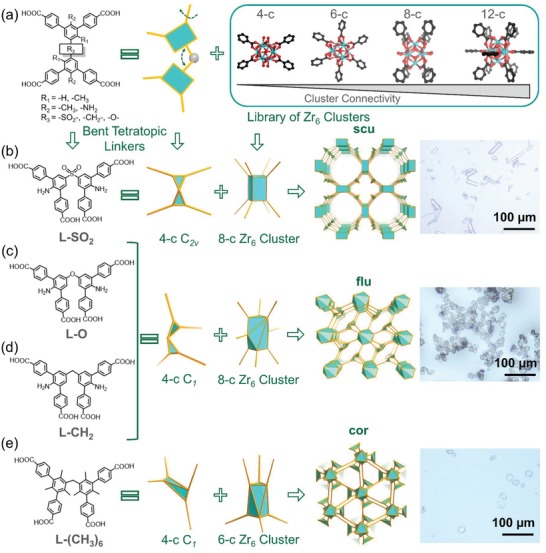
Illustration of various topologies in Zr‐tetracarboxylate frameworks based on bent linkers. a) The combination of bent tetratopic linkers and Zr_6_ clusters with varying connectivities (from 4‐c to 12‐c) leads to various topologies. b) The linking of 8‐connected Zr_6_ clusters with *C*_2v_ linkers (L‐SO_2_) leads to the formation of a **scu** net. c) Employment of a *C*_1_ 4‐c ligand (L‐O) prompted the formation of a novel 8‐c Zr_6_ cluster and its reticulation into a (4, 8)‐c **flu** net. d) Use of a *C*_1_ 4‐c ligand L‐CH_2_ resulted in the discovery of the same (4, 8)‐c **flu** net. e) The combination of 6‐c Zr_6_ clusters and 4‐c ligands with multiple bulky methyl groups (*C*_1_) generates a hierarchically assembled (4, 6)‐c **cor** net. Optical images of these Zr‐MOF single crystals were presented, showing varying morphologies of the corresponding structures.

Large colorless crystals, PCN‐901‐SO_2_ (also named UPC‐901‐SO_2_), were obtained with cuboid morphology after the solvothermal reactions between L‐SO_2_ and ZrCl_4_ in the presence of trifluoroacetic acid as a modulator for 24 h (Figure 1b). The structure of PCN‐901‐SO_2_ was determined by single‐crystal X‐ray diffraction. Powder X‐ray diffraction (PXRD) patterns indicate the phase purity of the obtained product (**Figure** **2**d). PCN‐901‐SO_2_ crystallized in the tetragonal space group P4/mmm (Table S1, Supporting Information). Crystallographically, it contains an 8‐connected Zr_6_ cluster [Zr_6_(µ_3_‐O)_4_(µ_3_‐OH)_4_(OH)_4_(H_2_O)_4_(COO)_8_] and a bent tetratopic linker with *C*_2v_ symmetry. The 8‐connected Zr_6_ clusters can be simplified as cuboid nodes, while the bent tetratopic linker can be viewed as 4‐connected joint triangular nodes (Figure 2a). The overall structure was analyzed to be a 4,8‐connected **scu** net with a point symbol of {4^16^.6^12^}{4^4^.6^2^}_2_ as determined by TOPOS 4.0 (Figure 2a).^22^ Note that this topology has been reported previously in NU‐901 and PCN‐606, which are constructed from rectangular planar nodes and cuboid nodes.^21^, ^23^ In the structure of PCN‐901‐SO_2_, the arrangement of Zr_6_ clusters leads to two types of pores (Figure 2a). Remarkably, the linkers are restricted into a conformation with 113.6° dihedral angle between the two central phenyl rings, and 82.9° dihedral angle between the two peripheral phenyl rings and the central ones. The chemical stability of PCN‐901‐SO_2_ was further examined by immersing MOFs in various aquesous solutions for 24 h. PXRD patterns indicated that the PCN‐901‐SO_2_ has excellent chemical stability in solution with pH ranging from 0 to 12 as evidenced by the well‐maintained crystallinity (Figure S14, Supporting Information).

**Figure 2:**
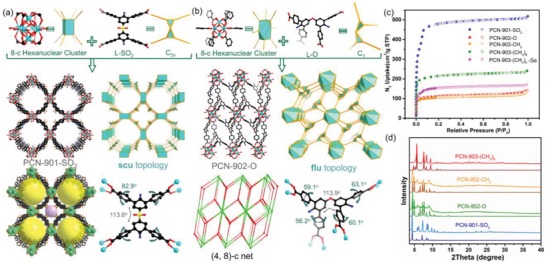
Structural illustration of PCN‐901‐SO_2_ with (4, 8)‐c scu topology and PCN‐902‐O with (4, 8)‐c flu topology. a) The assembly of 8‐c Zr_6_ clusters and 4‐c ligands with a SO_2_ substituent (*C*_2v_) into PCN‐901‐SO_2_ with a (4, 8)‐c **scu** net. b) The assembly of 8‐c Zr_6_ clusters and 4‐c ligands with an O substituent (*C*_1_) into PCN‐902‐O with a (4, 8)‐c **flu** net. c) N_2_ isotherms of PCN‐901(Zr)‐SO_2_, PCN‐902(Zr)‐O, PCN‐902(Zr)‐CH_2_, PCN‐903(Zr)‐(CH_3_)_6_, and PCN‐903(Zr)‐(CH_3_)_6_ after Se adsorption. d) Simulated and experimental Powder X‐ray diffraction (PXRD) patterns of PCN‐901(Zr)‐SO_2_, PCN‐902(Zr)‐O, PCN‐902(Zr)‐CH_2_, and PCN‐903(Zr)‐(CH_3_)_6_.

Interestingly, we observed large crystals of PCN‐902‐O with a different morphology formed after the reaction between ZrCl_4_ and L‐O under the same condition (Figure 1c). As indicated by single‐crystal X‐ray diffraction, L‐O possessed a *C*_1_ symmetry due to the bulkiness influence of the substituents (Figure 2b). Notably, the dihedral angle between the two central phenyl rings in L‐O is 113.9° and the dihedral angles between the central phenyl ring and the two peripheral ones are around 60°. The corresponding phase purity of the products was supported by PXRD analysis (Figure 2d). PCN‐902‐O crystallized in the triclinic space group *P*‐1 (Table S1, Supporting Information). Crystallographically, it contains an 8‐connected Zr_6_ cluster [Zr_6_(µ_3_‐O)_4_(µ_3_‐OH)_4_(OH)_4_(H_2_O)_4_(COO)_8_] and a 4‐connected bent tetratopic linker with *C*_1_ symmetry. The overall structure was determined to be a 4, 8‐connected **flu** net with a point symbol of {4^12^.6^12^.8^4^}{4^6^}_2_ by TOPOS 4.0 (Figure 2b). Replacing L‐O with a similar bent linker L‐CH_2_ generated the isostrucural PCN‐902‐CH_2_, as indicated by the PXRD pattern. The **flu** (fluorite) topology can be viewed as a cubic close packing (**ccp**) of the Ca^2+^ cations where the F^−^ anions occupy all tetrahedral interstitial cavities (Figure 2a), leaving all the octahedral interstitial cavities vacant. MOFs with **flu** topology can be assembled through 4‐connected linkers and 8‐connected metal clusters.

Additionally, introducing bulky methyl groups onto the phenyl rings of bent L‐CH_2_ can necessitate the two peripheral phenyl rings to exhibit a larger dihedral angle, leading to a different conformation for MOF construction. In order to obtain large single crystals, benzoic acid was used as the modulator to control the growth kinetics of PCN‐903‐(CH_3_)_6_, in which a truncated octahedron crystal with **cor** topology was built based upon an unprecedent 6‐c Zr_6_ cluster. Single‐crystal X‐ray analysis revealed that PCN‐903‐(CH_3_)_6_ crystallized in the trigonal space group *R‐3c* (**Figure** **3**). It contains a novel 6‐connected Zr_6_ cluster [Zr_6_(µ_3_‐O)_4_(µ_3_‐OH)_4_(OH)_6_(H_2_O)_6_(COO)_6_] and a tetratopic linker with *C*_1_ symmetry. Crystallographically, six bent tetratopic linkers bridged four neighboring Zr_6_ clusters, which can be simplified into a fascinating (4, 6)‐connected net with **cor** topology (Figure 3b).

**Figure 3:**
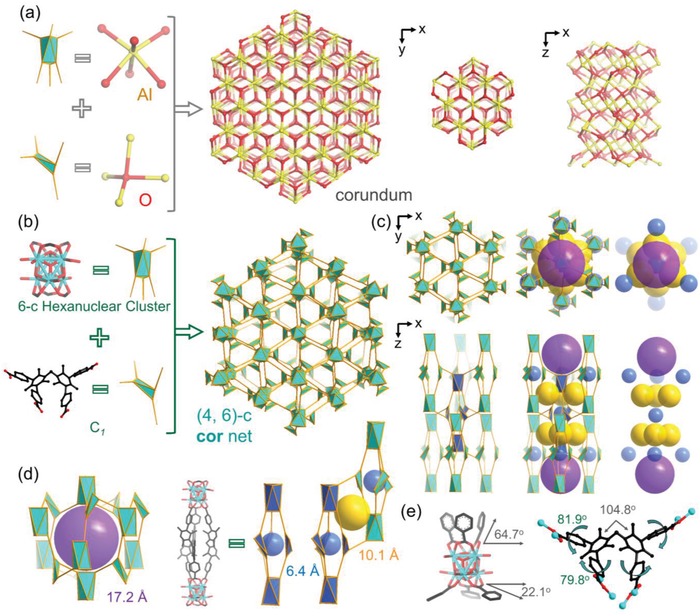
Structural illustration of PCN‐903‐(CH_3_)_6_ with (4, 6)‐c cor topology. a) Structural illustration of corundum (Al_2_O_3_) constructed from four‐connected O atoms and six‐connected Al atoms. b) The assembly of 6‐c Zr_6_ clusters and 4‐c ligands with bulky methyl substituents (*C*_1_) into a (4, 6)‐c **cor** net. c) The top and side views of PCN‐903‐(CH_3_)_6_, showing the connectivity modes of the building units and pore arrangement. d) PCN‐903‐(CH_3_)_6_ contains three types of pores with 6.4, 10.1, and 17.2 Å size. e) Illustration of the cluster and linker conformations in PCN‐903‐(CH_3_)_6_.

Different from the traditional 6‐connected Zr_6_ cluster observed in MOF‐808 and other 6‐c Zr‐MOFs, the Zr_6_ cluster in PCN‐903‐(CH_3_)_6_ displays two types of dihedral angles, 64.7° and 22.1°, between the O–Zr–Zr–O plane and the equatorial plane (Figure 3e). This unusual asymmetric feature of Zr_6_ clusters brings new opportuities to explore topology diversity within highly connected MOFs. The overall structure was analyzed to be a (4, 6)‐connected net with a point symbol of {4^3^.6^3^}_3_{4^6^.6^9^}_2_ as determined by TOPOS 4.0 (Figure 3b).

The intriguing **cor** topology originates from the lattice of corundum, a crystalline phase of aluminium oxide (Al_2_O_3_), where each Al atom is surrounded by six O atoms.^24^ In the corundum lattice, the O atoms never locate at the corners of a regular octahedron, instead, they form a slightly distorted hexagonal close packing, where two‐thirds of octahedral interstitial cavities are occupied by Al atoms. By replacing Al atoms with distorted 6‐connected Zr clusters and O atoms with bent tetratopic ligands, the extension from an inorganic material to a porous inorganic–organic hybrid compound is achieved (Figure 3a). The assembly of 6‐c Zr_6_ clusters and *C*_1_ ligands initially generates a supramolecular triangular bipyramid building unit, where all equatorial vertices are occupied by the ligand building units while both polar vertices are placed by the inorganic clusters (Figure 3d). As a result, a microporous cage with 6.4 Å size is formed. Further hierarchical assembly of these triangular bipyramid building units into 3D network can be visualized in Figure 3. Each triangular bipyramid unit is connected to neighboring six units, resulting in a second type of pore with 10.1 Å size between each two triangular bipyramid units. Further assembly of these units also generates a larger cage with 17.2 Å size (Figure 3c). The discovery of PCN‐903‐(CH_3_)_6_ presents a very rare case, and a new level of sophisticated assembly of Zr‐cluster based MOFs involving multiple levels of structural hierarchy. It should be noted that there is a β‐UH_3_‐like topology based Zr‐MOF constructed from 4‐connected elongated linkers reported by Zhang and co‐workers.^25^ Our work here presents a powerful strategy to ascertain new structural mysteries of MOFs which mimic complicated inorganic structures. In addition, there is also a Zn‐MOF, Zn_4_O(BenzTB)_3/2_ (DUT‐13) with very high pore volume, exhibiting **cor** topology.^26^

The permanent porosity of the PCN‐90X(Zr) series was confirmed by N_2_ sorption isotherms measured at 77 K (Figure 2c). PCN‐90X samples were washed thoroughly using *N*,*N*‐dimethylformamide (DMF), CH_2_Cl_2_ and hexane for several times before gas sorption measurements. PCN‐90X(Zr) series exhibit a typical type I isotherm, suggesting microporosity. Due to the densely packed building blocks, PCN‐902 and PCN‐903 showed relatively low N_2_ uptake and surface areas.

The effects of center functional groups on linker conformation have been explored through density functional theory calculations. The relative energies of selected linkers in PCN‐901, PCN‐902, and PCN‐903 were calculated and listed in **Figure** **4** and Table S2 (Supporting Information). For clarify, the energies of a given linker fragment in different conformations were normalized by subtracting the energy of its unconstrained structure. Due to the constraints from Zr_6_ clusters in the frameworks, two lateral phenyl rings in the individual ligand will exhibit distinct torsion angles in the corresponding structures with different topologies. For linker L‐SO_2_, the linker is too rigid to distort, making the formation of conformations observed in PCN‐902 and PCN‐903 unfavorable. The energy difference of L‐O in PCN‐901 and PCN‐902 is ≈20.6 kJ mol^−1^, while the difference is ≈14.4 kJ mol^−1^ for L‐O in PCN‐902 and PCN‐903. This result indicates that the bent conformation of L‐O in PCN‐902 allows for the gyration of two lateral phenyl rings, which is beneficial for the formation of PCN‐902 with **flu** topology. For L‐CH_2_ with a similar conformation, the formation of **flu** topology is also favorable as indicated by the relative energies in PCN‐90X with varying topologies. Considering the linker L‐(CH_3_)_6_, its conformation is latched by bulky methyl groups. By comparing the relative energies of linkers in PCN‐90X, we observed that ligands with bulky groups such as —CH_3_ tend to be obtained with a larger torsion angle, so only PCN‐903 with **cor** topology will be adapted in this case.

**Figure 4:**
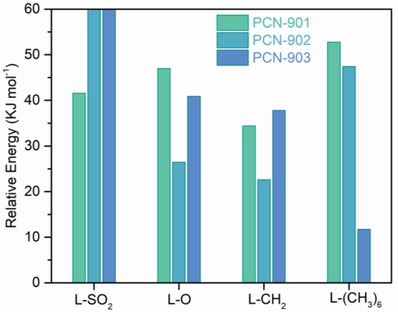
Relative energies of ligands with various conformations in PCN‐90X series possessing different topologies. The relative energy for a given ligand is normalized through subtracting the energy of its unconstrained structure.

Due to the existing large amount of coordinately unsaturated sites on Zr_6_ clusters, the highly defective PCN‐903‐(CH_3_)_6_ was tested for its ability to adsorb and remove selenite anions from aqueous solutions. Although trace amounts of selenium are vital for nutritional needs of animals, it becomes extremely toxic at high concentrations (>400 µg d^−1^).^27^, ^28^, ^29^ The maximum acceptable concentrations were set as 40 and 50 ppb, respectively, by the World Health Organization (WHO) and the United States Environmental Protection Agency (USEPA).^30^ The uptake ability of PCN‐903‐(CH_3_)_6_ toward toxic SeO_3_^2−^ was first tested via a series of SeO_3_^2−^ aqueous solution from 10 to 500 ppm. To reach adsorption equilibrium, 10 mg PCN‐903‐(CH_3_)_6_ was immersed in the aqueous solutions for 24 h, which was followed by inductively coupled plasma mass spectrometry (ICP‐MS) analysis of supernatant fraction to determine the remaining SeO_3_^2−^ concentration. As indicated by **Figure** **5**a, the adsorb amounts of SeO_3_^2−^ in the MOF absorbent increase as the initial concentrations of SeO_3_^2−^ increase. The maximum Se capacity of PCN‐903‐(CH_3_)_6_ was calculated as 75.5 mg g^−1^, which exhibits one of the highest adsorption capacities among MOF absorbents (Figure 5a and Table S4, Supporting Information).^30^, ^31^ The kinetic behavior for SeO_3_^2−^ was subsequently investigated (Figure 5b and Table S5, Supporting Information). Remarkably, PCN‐903‐(CH_3_)_6_ exhibits rapid removal, >90% of SeO_3_^2−^ within 7 min, >99% removal within 1 h and complete removal, >99.9%, of SeO_3_^2−^ within 3 h. The excellent selenite removal efficiency places PCN‐903‐(CH_3_)_6_ as a promising porous absorbent for detoxification.

**Figure 5:**
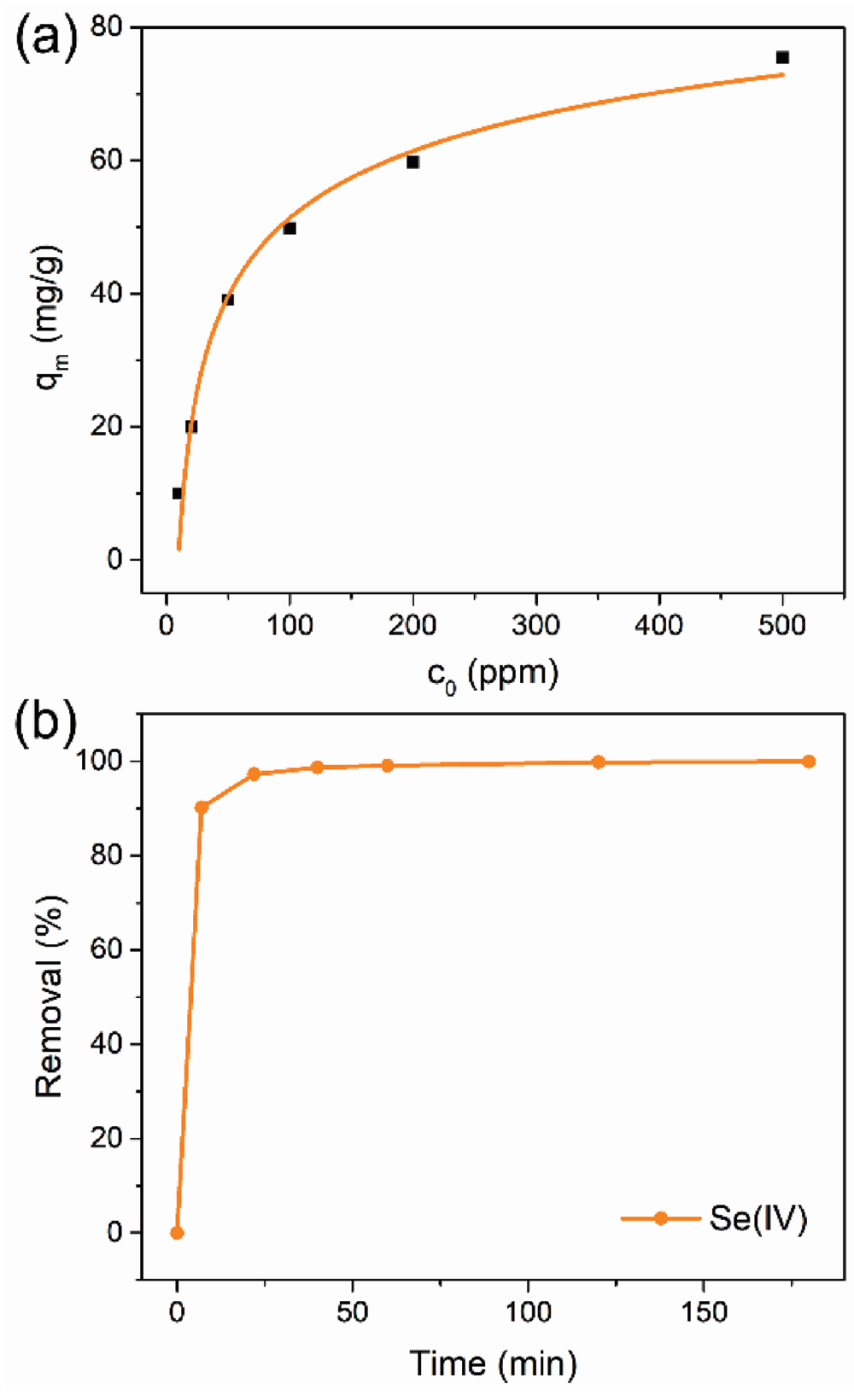
Efficient removal of toxic selenite ions by PCN‐903‐(CH_3_)_6_. a) Sorption isotherms for selenite (SeO_3_^2−^) by PCN‐903‐(CH_3_)_6_. b) Sorption kinetics curves for selenite (SeO_3_^2−^) by PCN‐903‐(CH_3_)_6_.

In conclusion, we have synthesized four new Zr‐MOFs based on desymmetrized tetratopic linkers with various substituents. For example, PCN‐901‐SO_2_ is constructed from the assembly between a bent linker L‐SO_2_ with *C*_2v_ symmetry and an 8‐connected Zr_6_ cluster; PCN‐902‐O/CH_2_ contains a novel 8‐connected Zr_6_ cluster and a bent linker L‐O/‐CH_2_ with *C*_1_ symmetry, exhibiting a CaF_2_‐like **flu** topology; further utilization of restricted bent linker [(L‐(CH_3_)_6_)] gives rise to PCN‐903‐(CH_3_)_6_ with a fascinating Al_2_O_3_‐like (4, 6)‐c **cor** net. Additionally, the highly defective PCN‐903‐(CH_3_)_6_ showed efficient removal of toxic selenite ions in aqueous solutions, achieving >90% of SeO_3_^2−^ within 7 min, and complete removal, >99.9%, of SeO_3_^2−^ within 3 h. This work points out a fresh direction for developing functional and stable Zr‐MOFs with unprecedented topologies by linker design.

## Experimental Section

*Linker Synthesis*: The detailed synthetic approaches of organic linkers including L‐SO_2_, L‐CH_2_, L‐SO_2_, and L‐(CH_3_)_6_, are provided in the Supporting Information.

*Synthesis of PCN‐901(Zr)‐SO_2_*: ZrCl_4_ (20 mg), L‐SO_2_ (10 mg), and DMF (3 mL) were charged into a 10 mL vial, followed by the addition of 0.15 mL trifluoroacetic acid. The mixture was heated in 115 °C oven for 2 d. After cooling down to room temperature, the colorless crystals of PCN‐901(Zr)‐SO_2_ were harvested (yield: 75%).

*Synthesis of PCN‐902(Zr)‐O*: ZrCl_4_ (20 mg), L‐O (10 mg), and DMF (3 mL) were charged into a 10 mL vial, followed by the addition of 0.10 mL trifluoroacetic acid. The mixture was heated in 120 °C oven for 2 d. After cooling down to room temperature, the colorless crystals of PCN‐902(Zr)‐O were harvested (yield: 70%).

*Synthesis of PCN‐902(Zr)‐CH_2_*: ZrCl_4_ (20 mg), L‐CH_2_ (10 mg), and DMF (3 mL) were charged into a 10 mL vial, followed by the addition of 0.10 mL trifluoroacetic acid. The mixture was heated in 120 °C oven for 2 d. After cooling down to room temperature, the colorless crystals of PCN‐902(Zr)‐CH_2_ were harvested (yield: 71%).

*Synthesis of PCN‐903(Zr‐CH_3_)_6_*: ZrCl_4_ (20 mg), L‐(CH_3_)_6_ (10 mg), benzoic acid (500 mg), and DMF (3 mL) were charged into a 10 mL vial. The mixture was heated in 120 °C oven for 2 d. After cooling down to room temperature, the colorless crystals of PCN‐903(Zr‐CH_3_)_6_ were harvested (yield: 62%).

*Adsorption Isotherm Measurement*: PCN‐903‐(CH_3_)_6_ (10 mg) was mixed with a 10 mL stock solution of selenite (SeO_3_^2−^) with various concentrations. The mixture was kept at room temperature for 24 h to ensure complete adsorption. The ion concentration in the supernatant solutions was determined by ICP‐MS.

*Adsorption Kinetics Measurement*: PCN‐903‐(CH_3_)_6_ (10 mg) was mixed with a 10 mL stock solution of selenite (SeO_3_^2−^) at a concentration of 10 ppm. The mixture was kept at room temperature for various periods. During the adsorption process, the ion concentration in the supernatant solutions was analyzed by ICP‐MS.

## Conflict of Interest

The authors declare no conflict of interest.

## Supporting information

Supplementary
