# Supplementary material for: Uncovering Structural Opportunities for Zirconium Metal–Organic Frameworks via Linker Desymmetrization
Source: Adv Sci (Weinh). 2019 Sep 30;6(23):1901855. doi: 10.1002/advs.201901855 (PMC6891898; doi:10.1002/advs.201901855)
Supplement: Supplementary file 1 — Supplementary [file ADVS-6-1901855-s001.pdf]

Copyright WILEY-VCH Verlag GmbH & Co. KGaA, 69469 Weinheim, Germany, 2019.

ADVANCED SCIENCE
Open Access

Supporting Information

for *Adv. Sci.,* DOI: 10.1002/advs.201901855

Uncovering Structural Opportunities for Zirconium Metal–Organic Frameworks via Linker Desymmetrization

Yutong Wang, Liang Feng, Kai Zhang, Kun-Yu Wang, Weidong Fan, Xiaokang Wang, Bingbing Guo, Fangna Dai, Liangliang Zhang, Daofeng Sun, and Hong-Cai Zhou**

Supporting Information

Uncovering Structural Opportunities for Zirconium Metal–Organic Frameworks via Linker Desymmetrization

Yutong Wang[†,‡], Liang Feng [§,‡], Kai Zhang[†], Kun-Yu Wang[§], Weidong Fan[†], Xiaokang Wang[†], Bingbing Guo[†], Fangna Dai[†], Liangliang Zhang[‖], Daofeng Sun[†]*, and Hong-Cai Zhou[§#]*

[†] College of Science, School of Materials Science and Engineering, China University of Petroleum (East China), Qingdao, Shandong 266580, China

[§] Department of Chemistry, Texas A&M University, College Station, Texas 77843-3255, United States

[#] Department of Materials Science and Engineering, Texas A&M University, College Station, Texas 77843-3003, United States

‖ Xi'an Institute of Flexible Electronics, Northwestern Polytechnical University, Xi'an 710072, China

[‡] These authors contributed equally to this work.

Corresponding Authors:

*dfsun@upc.edu.cn

*zhou@chem.tamu.edu

Contents

S1. Ligand Synthesis 3

S2. MOF Synthesis 12

S3. Single Crystal X-ray Crystallography 14

S4. Computational Methods 16

S5. Powder X-ray Diffraction..19

S6. Gas Sorption Isotherm...21

S7. Thermogravimetric Analysis 24

S8. Removal of Toxic Selenite Ions..26

S1. Ligand Synthesis

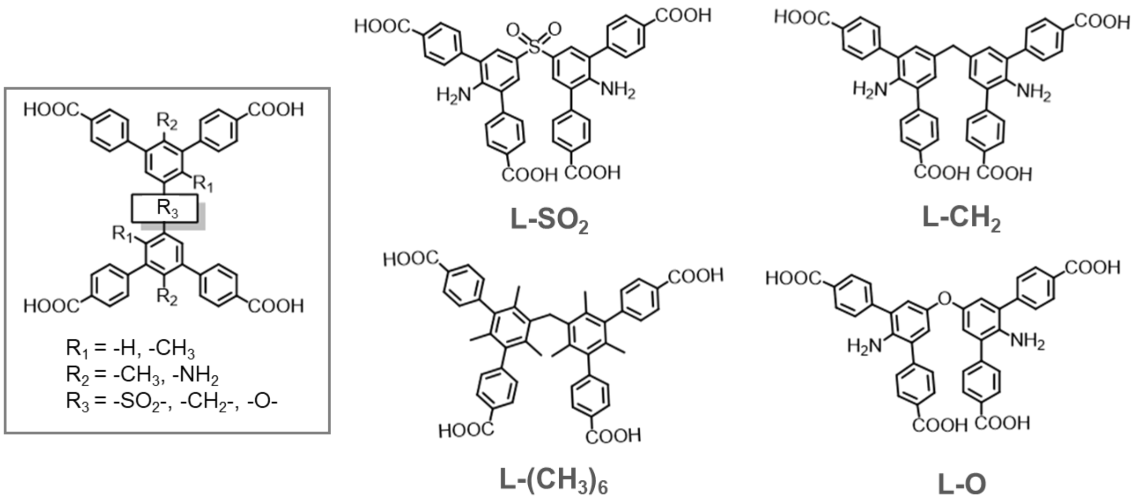

Figure S1. Tetratopic linkers with various pivot hinges in this work, **L-SO_2, L-CH_2, L-$(CH_3)_6$** and **L-O.**

Synthesis of L-SO_2

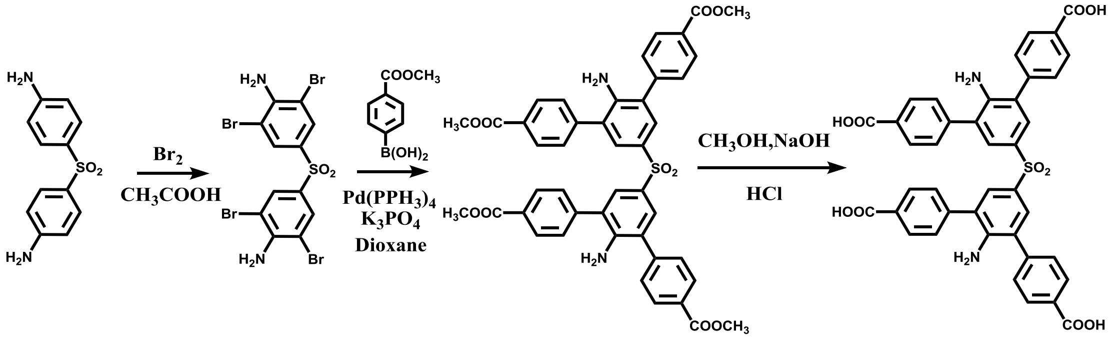

Scheme S1. Synthetic procedure for **L-SO_2**.

(1) 4,4'-sulfonylbis(2,6-dibromoaniline)

4,4'-sulfonyldianiline (2.48 gm, 0.01 mole) was dissolved in glacial acetic acid (15 ml), followed by the addition of bromine in acetic acid (32ml, 0.01 mol, 20% bromine in acetic acid). The mixture was kept stirred overnight at room temperature, followed by dilution with excess cold water. The solid product was filtered, washed with cold water, dried and recrystallized from ethyl acetate. Yield 85%. ^{1}H NMR (400 MHz, DMSO-d6): δ 6.28-7.97 (s, 4H), 3.32(s,2H) ppm. Anal. Calc. for $C_{12}H_8Br_4N_2O_2S$: N,4.97, Br,56.68 Found: N, 5.03, Br, 56.89.[1]

(2) Tetramethyl 5',5''''-sulfonylbis(2'-amino-[1,1':3',1''-terphenyl]-4,4''-dicarboxylate)

4,4'-sulfonylbis(2,6-dibromoaniline) (2.82 g, 5 mmol), methyl 4-boronobenzoate (4.32 g, 24 mmol), Pd$(PPh_3)_4$ (0.3 g, 0.26 mmol) and K_3PO_4 (10.64 g, 40 mmol) were

placed in a 500 ml two-necked round bottom flask under N_2 atmosphere. The flask was further charged with 200 mL dry 1,4-dioxane, and heated for 48 h. After cooling down to room temperature, the solvent was removed followed by the addition of water. The water phase was washed with CH_2Cl_2. The mixed organic phase was then dried by $MgSO_4$. After the solvent was removed, the crude product was purified by column chromatography with CH_2Cl_2 as the eluent. Yield 90%. ^{1}H NMR (400 MHz, $CDCl_3$) δ 3.95(s, 12H), 7.53(d, 8H), 7368(s, 4H), 8.15(d, 8H) ppm. Anal. Calc. for $C_{44}H_{36}N_2O_{10}S$ (mw 784.84): C, 67.34; H, 4.62; N, 3.57; O, 20.39; S, 4.08. Found: C, 67.27; H, 4.55; N, 3.47; S, 4.18.

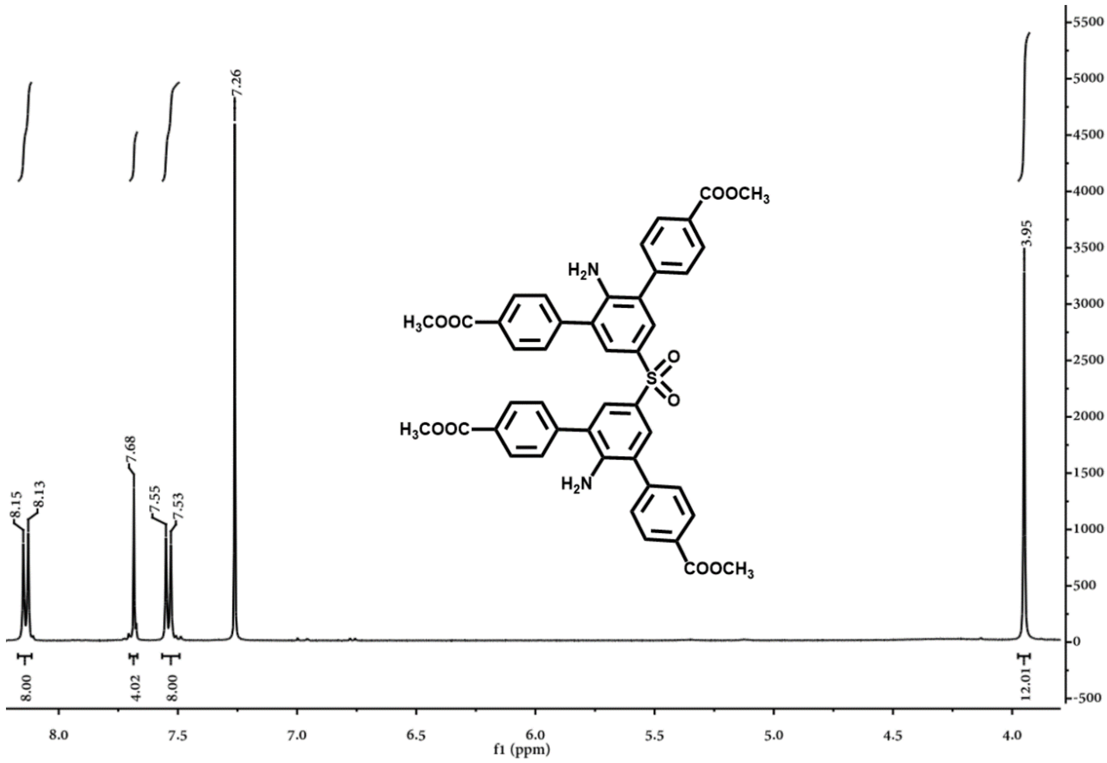

Figure S2. ^{1}H NMR of Tetramethyl 5',5''''-sulfonylbis(2'-amino-[1,1':3',1''-terphenyl]-4,4''-dicarboxylate).

(3) 5',5''''-sulfonylbis(2'-amino-[1,1':3',1''-terphenyl]-4,4''-dicarboxylic acid)

Tetramethyl 5',5''''-sulfonylbis(2'-amino-[1,1':3',1''-terphenyl]-4,4''-dicarboxylate) (3.92 g, 5 mmol) was dissolved in 50 mL MeOH followed by the addition of 50 mL 2 M NaOH aqueous solution. The mixture was stirred at 50 °C overnight. The organic phase was removed, while the aqueous phase was acidified with diluted hydrochloric acid. The yellow precipitate was further filtered and washed with water for several times. Yield 98%. ^{1}H NMR (400 MHz, DMSO-d6) δ 8.05(d, 8H), 7.56-7.59(m, 12H), 5.25(s, 4H) ppm. Anal. Calc. for $C_{40}H_{28}N_2O_{10}S$ (mw 728.73): C, 65.93; H, 3.87; N, 3.84; O, 21.95; S, 4.40. Found: C, 65.78; N, 3.73; H, 4.00; S, 4.02.

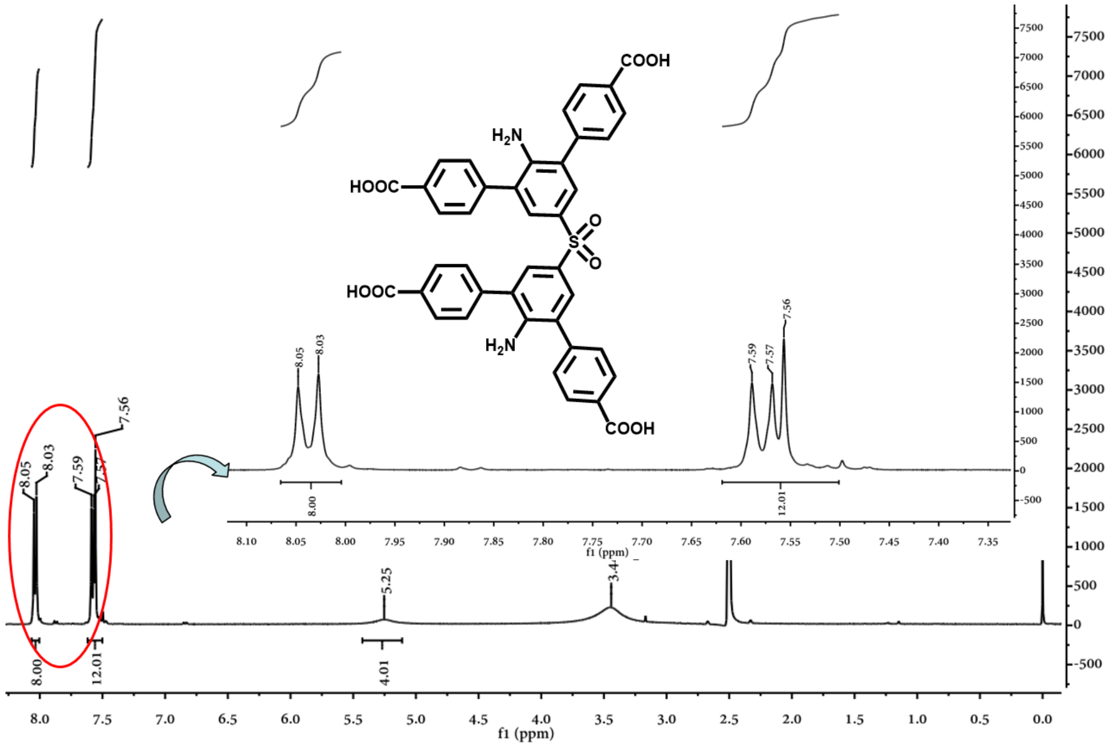

Figure S3. ^{1}H NMR of **L-SO$_2$**.

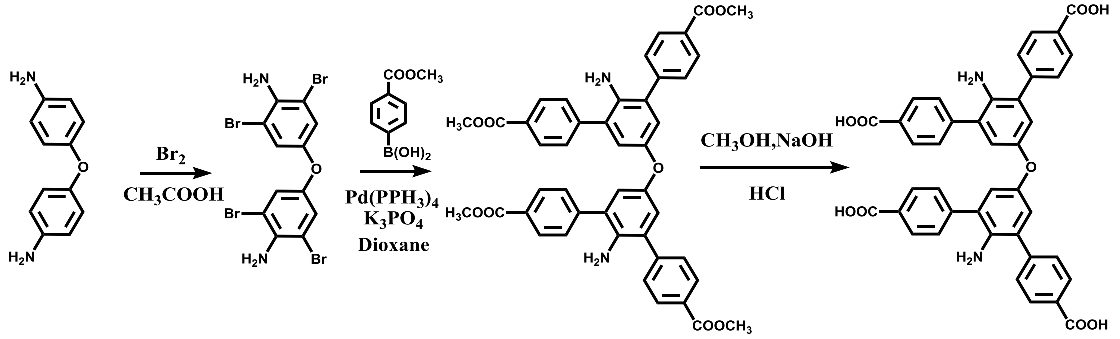

Scheme S2. Synthetic procedure for **L-O**.

(1) 4,4'-oxybis(2,6-dibromoaniline)

Bromine (2.0 mL, 40 mmol) was added to 4,4'-diaminodiphenyl ether (2.0 g, 10 mmol) in acetic acid (50 mL) at room temperature. The reaction was stirred at 35 °C for 5 min and then poured into cold water (200 mL). The crude product was filtered off and purified on silica gel column with n-hexane/ethyl acetate (6:1) as the mobile phase. Yield: 3.7 g, 7.2 mmol, 72%.^{1}H NMR ($CDCl_3$) δ 7.07 (s, 4H) ppm, 4.45 (s, 4H) ppm. Anal. Calc. for $C_{12}H_8Br_4N_2O$ (mw 515.83): C, 27.94; H, 1.56; Br, 61.96; N, 5.43; O, 3.10. Found: C, 27.78; N, 5.73; H, 1.23; Br, 62.03.[2]

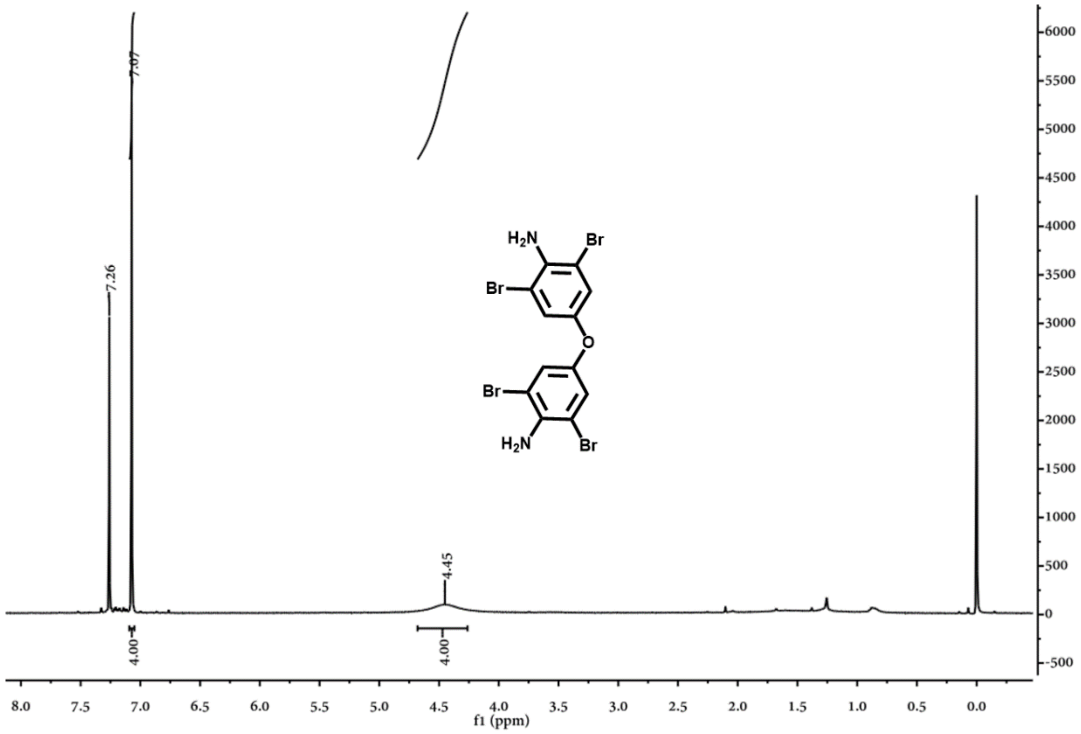

Figure S4. ^{1}H NMR of 4,4'-oxybis(2,6-dibromoaniline).

(2) Tetramethyl 5',5''''-oxybis(2'-amino-[1,1':3',1''-terphenyl]-4,4''-dicarboxylate)

4,4'-oxybis (2,6-dibromoaniline) (2.56 g, 5 mmol), methyl 4-boronobenzoate (4.32 g, 24 mmol), $Pd(PPh_3)_4$ (0.3 g, 0.26 mmol) and K_3PO_4 (10.64 g, 40 mmol) were placed in a 500 ml two-necked round bottom flask under N_2 atmosphere. The flask was further charged with 200 mL dry 1,4-dioxane, and heated for 48 h. After cooling down to room temperature, the solvent was removed followed by the addition of water. The water phase was washed with CH_2Cl_2. The mixed organic phase was then dried by $MgSO_4$. After the solvent was removed, the crude product was purified by column chromatography with CH_2Cl_2 as the eluent. Yield 86%. ^{1}H NMR (400 MHz, $CDCl_3$) δ 3.94(s, 12H), 6.91(s, 4H), 7.56(d, 8H), 8.11(d, 8H) ppm. Anal. Calc. for $C_{44}H_{36}N_2O_9$ (mw 736.24): C, 71.73; H, 4.93; N, 3.80; O, 19.54. Found: C, 71.27; H, 4.55; N, 3.47.

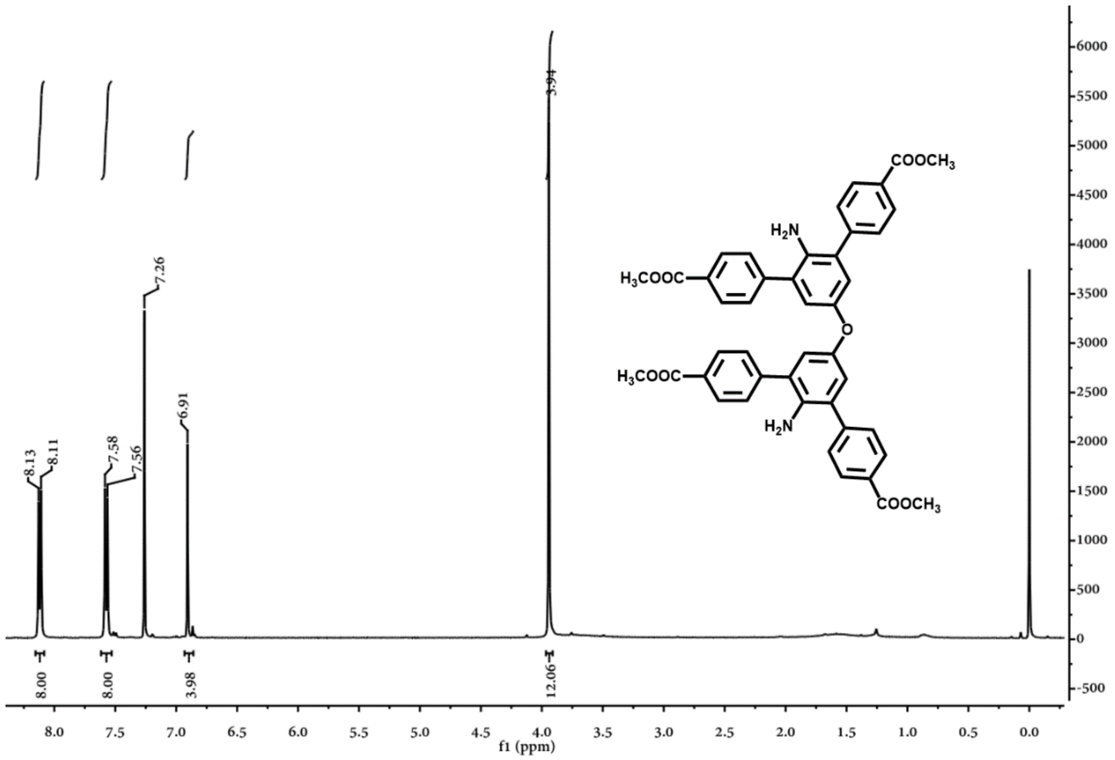

Figure S5. ^{1}H NMR of tetramethyl 5',5''''-oxybis(2'-amino-[1,1':3',1''-terphenyl]-4,4''-dicarboxylate).

(3) 5',5''''-oxybis(2'-amino-[1,1':3',1''-terphenyl]-4,4''-dicarboxylic acid)

Tetramethyl 5',5''''-oxybis(2'-amino-[1,1':3',1''-terphenyl]-4,4''-dicarboxylate) (3.68 g, 5 mmol) was dissolved in 50 mL MeOH, followed by the addition of 50 mL 2 M NaOH aqueous solution. The mixture was stirred at 50 °C overnight. The organic phase was removed, and the aqueous phase was acidified with diluted hydrochloric acid. The yellow precipitate was filtered and washed with water for several times. Yield 98%. ^{1}H NMR (400 MHz, DMSO-d6) δ 12.99 (s, 4H), 8.02(d, 8H), 7.61(d, 8H), 6.86(d, 4H) ppm. Anal. Calc. for $C_{40}H_{28}N_2O_9$ (mw 680.67): C, 70.58; H, 4.15; N, 4.12; O, 21.15. Found: C, 70.78; N, 3.93; H, 4.07.

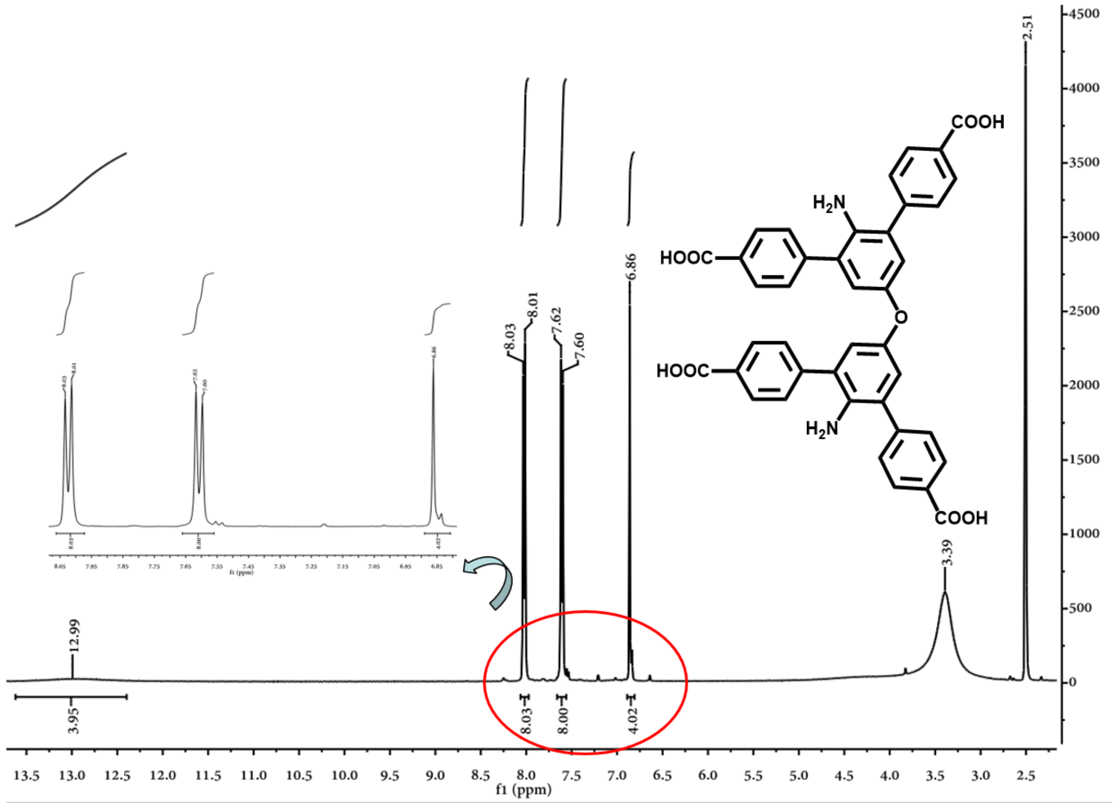

Figure S6. ^{1}H NMR of **L-O**.

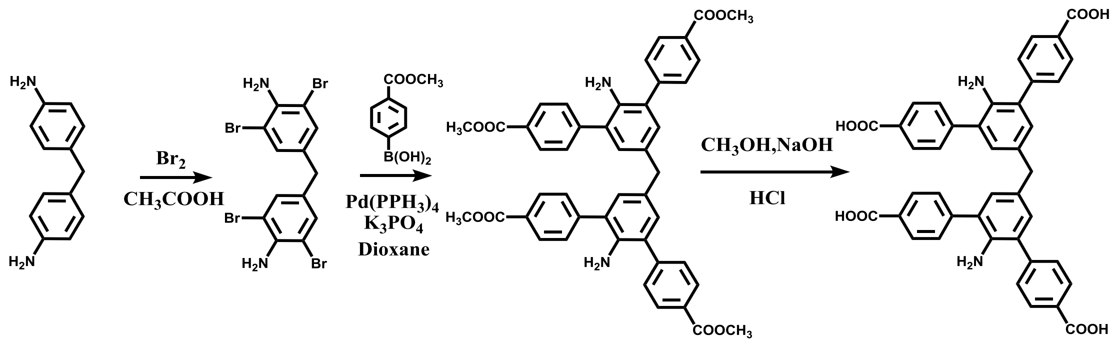

Scheme S3. Synthetic procedure for **L-CH$_2$**.

(1) 4,4'-methylenebis(2,6-dibromoaniline)

Bromine (2.0 mL, 40 mmol) was added to 4,4'-methylenedianiline (1.98 g, 10 mmol) in acetic acid (50 mL) at room temperature. The reaction was stirred at 35 °C for 5 min and then poured into cold water (200 mL). It was filtered, washed with cold water, dried and crystallized from ethyl acetate. Yield: 3.85 g, 7.5 mmol, 75%. ^{1}H NMR ($CDCl_3$) δ 7.16 (s, 4H) ppm, 3.66 (s, 2H) ppm. Anal. Calc. for $C_{13}H_{10}Br_4N_2$ (mw 513.85): C, 30.39; H, 1.96; Br, 62.20; N, 5.45. Found: C, 29.78; N, 5.33; H, 1.53; Br, 62.33.

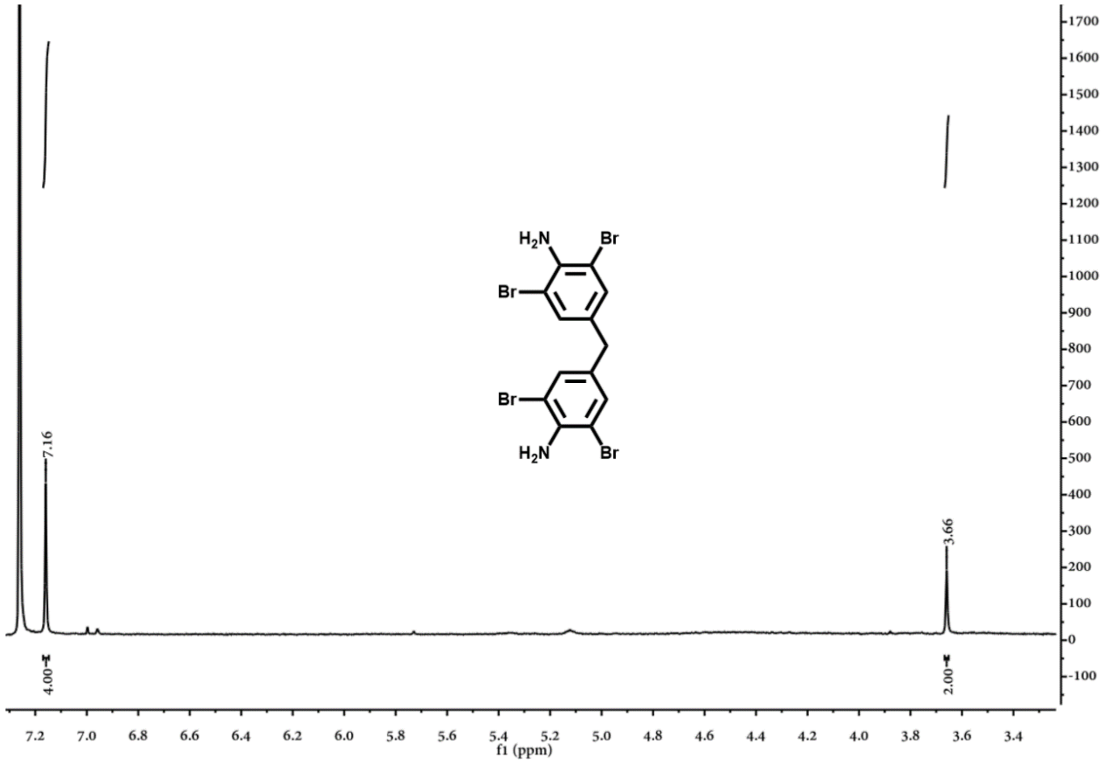

Figure S7. ^{1}H NMR of 4,4'-methylenebis(2,6-dibromoaniline).

(2) Tetramethyl 5',5''''-methylenebis(2'-amino-[1,1':3',1''-terphenyl]-4,4''-dicarboxylate)

4,4'-methylenebis(2,6-dibromoaniline) (2.56 g, 5 mmol), Methyl 4-boronobenzoate (4.32 g, 24 mmol), $Pd(PPh_3)_4$ (0.3 g, 0.26 mmol) and K_3PO_4 (10.64 g, 40 mmol) were placed in a 500 ml two-necked round bottom flask under N_2 atmosphere. The flask was further charged with 200 mL dry 1,4-dioxane, and heated for 48 h. After cooling down to room temperature, the solvent was removed followed by the addition of water. The water phase was washed with CH_2Cl_2. The mixed organic phase was then dried by $MgSO_4$. After the solvent was removed, the crude product was purified by column chromatography with CH_2Cl_2 as the eluent. Yield 88%. ^{1}H NMR (400 MHz, $CDCl_3$) δ 3.91(s, 2H), 3.94(s, 12H), 7.02(s, 4H), 7.56(d, 8H), 8.11(d, 8H) ppm. Anal. Calc. for $C_{45}H_{38}N_2O_8$ (mw 734.81): C, 73.56; H, 5.21; N, 3.81; O, 17.42. Found: C, 73.27; H, 5.55; N, 3.57.

(3) 5',5''''-methylenebis(2'-amino-[1,1':3',1''-terphenyl]-4,4''-dicarboxylic acid)

Tetramethyl 5',5''''-methylenebis(2'-amino-[1,1':3',1''-terphenyl]-4,4''-dicarboxylate) (3.67 g, 5 mmol) was dissolved in 50 mL MeOH followed by the addition of 50 mL 2 M NaOH aqueous solution. The mixture was stirred at 50 °C overnight. The organic phase was removed, while the aqueous phase was acidified with diluted hydrochloric acid. The yellow precipitate was further filtered and washed with water for several

times. Yield 98%. ^{1}H NMR (400 MHz, DMSO-d6) δ 8.02 (d, 8H), 7.59(d, 8H), 7.07(d, 4H) ppm. Anal. Calc. for $C_{41}H_{30}N_2O_8$ (mw 678.70): C, 72.56; H, 4.46; N, 4.13; O, 18.86. Found: C, 71.78; N, 3.98; H, 4.27.

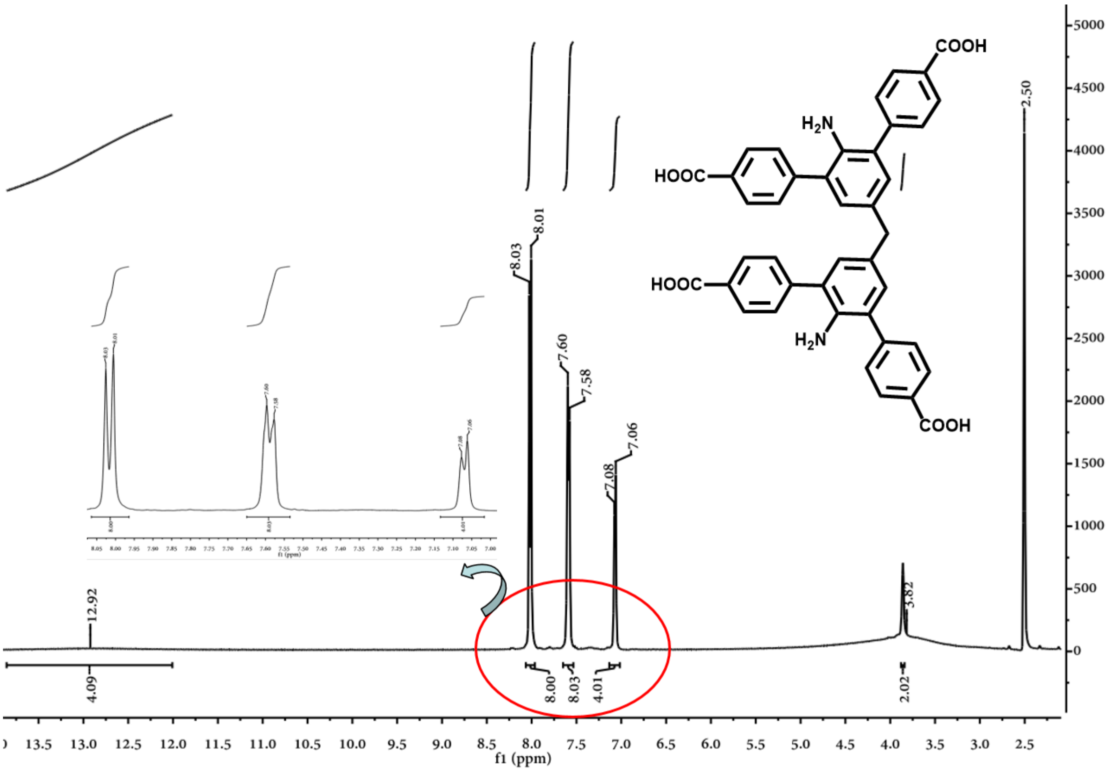

Figure S8. ^{1}H NMR of **L-CH$_2$**.

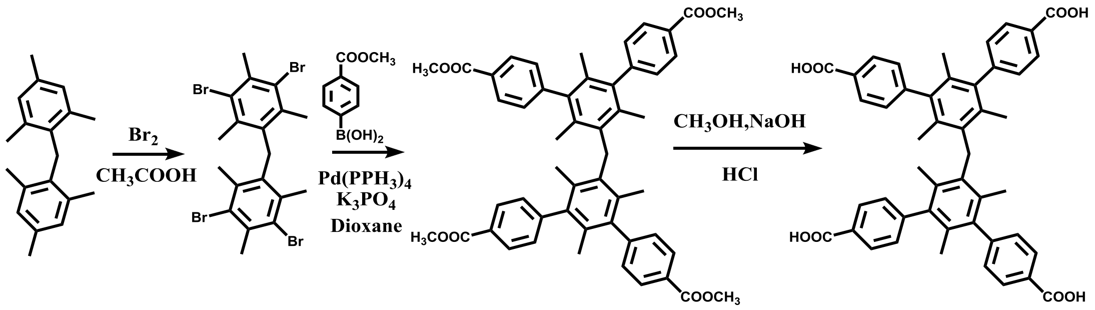

Scheme S4. Synthetic procedure for **L-(CH$_3$)$_6$**.

(1) Bis(3,5-dibromo-2,4,6-trimethylphenyl)methane

Bromine (2.0 mL, 40 mmol) was added to dimesitylmethane (2.52 g, 10 mmol) in acetic acid (50 mL) at room temperature. The reaction was stirred at 35 °C for 5 min and then poured into cold water (200 mL). The crude product was filtered off and purified on silica gel column with n-hexane/ethyl acetate (6:1) as the mobile phase. Yield: 3.85 g, 7.5 mmol, 75%.^{1}H NMR ($CDCl_3$) δ 1.45(s,6H), 1.76(s,12H), 4.17(s, 2H) ppm. Anal. Calc. for $C_{19}H_{20}Br_4$ (mw 567.99): C, 40.18; H, 3.55; Br, 56.27. Found: C, 40.59; H, 3.53; Br, 55.73.

(2) Tetramethyl 5',5''''-methylenebis(2',4',6'-trimethyl-[1,1':3',1''-terphenyl]-4,4''-dicarboxylate)

Bis(3,5-dibromo-2,4,6-trimethylphenyl)methane (2.84 g, 5 mmol), Methyl 4-boronobenzoate (4.32 g, 24 mmol), $Pd(PPh_3)_4$ (0.3 g, 0.26 mmol) and K_3PO_4 (10.64 g, 40 mmol) were placed in a 500 ml two-necked round bottom flask under N_2 atmosphere. The flask was further charged with 200 mL dry 1,4-dioxane, and heated for 48 h. After cooling down to room temperature, the solvent was removed followed by the addition of water. The water phase was washed with CH_2Cl_2. The mixed organic phase was then dried by $MgSO_4$. After the solvent was removed, the crude product was purified by column chromatography with CH_2Cl_2 as the eluent. Yield 87%. ^{1}H NMR (400 MHz, $CDCl_3$) δ 1.55(s,6H), 1.86(s,12H), 3.94(s, 12H), 4.27(s, 2H), 7.20(d, 8H), 8.10(d, 8H) ppm. Anal. Calc. for $C_{51}H_{48}O_8$ (mw 788.94): C, 77.64; H, 6.13; O, 16.22. Found: C, 77.27; H, 6.55.

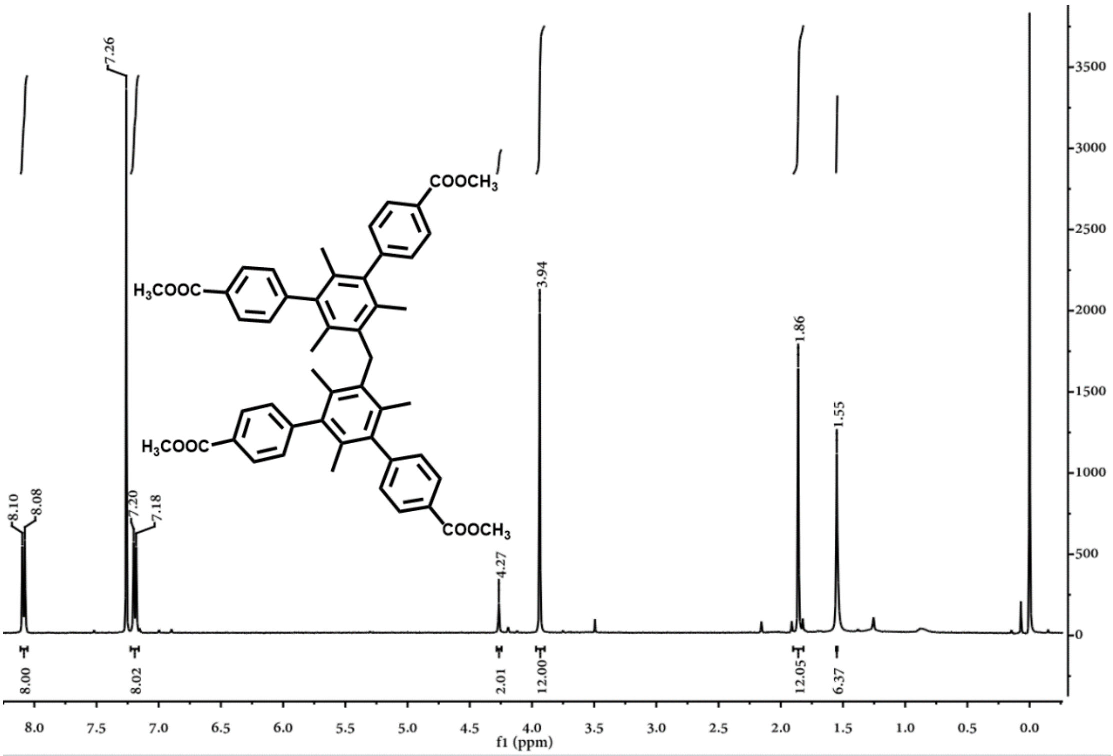

Figure S9. ^{1}H NMR of tetramethyl 5',5''''-methylenebis(2',4',6'-trimethyl-[1,1':3',1''-terphenyl]-4,4''-dicarboxylate).

(3) 5',5''''-methylenebis(2',4',6'-trimethyl-[1,1':3',1''-terphenyl]-4,4''-dicarboxylic acid)

Tetramethyl 5',5''''-methylenebis(2',4',6'-trimethyl-[1,1':3',1''-terphenyl]-4,4''-dicarboxylate) (3.94 g, 5 mmol) was dissolved in 50 mL MeOH followed by the addition of 50 mL 2 M

NaOH aqueous solution. The mixture was stirred at 50 °C overnight. The organic phase was removed, while the aqueous phase was acidified with diluted hydrochloric acid. The yellow precipitate was further filtered and washed with water for several times. Yield 98%. ^{1}H NMR (400 MHz, DMSO-d6) δ 12.93 (s, 4H), 8.01 (d, 8H), 7.22 (d, 8H), 4.24 (s, 2H), 1.81 (m, 12H) ppm. Anal. Calc. for $C_{41}H_{30}N_2O_8$ (mw 732.83): C, 77.03; H, 5.50; O, 17.47. Found: C, 77.78; H, 5.27.

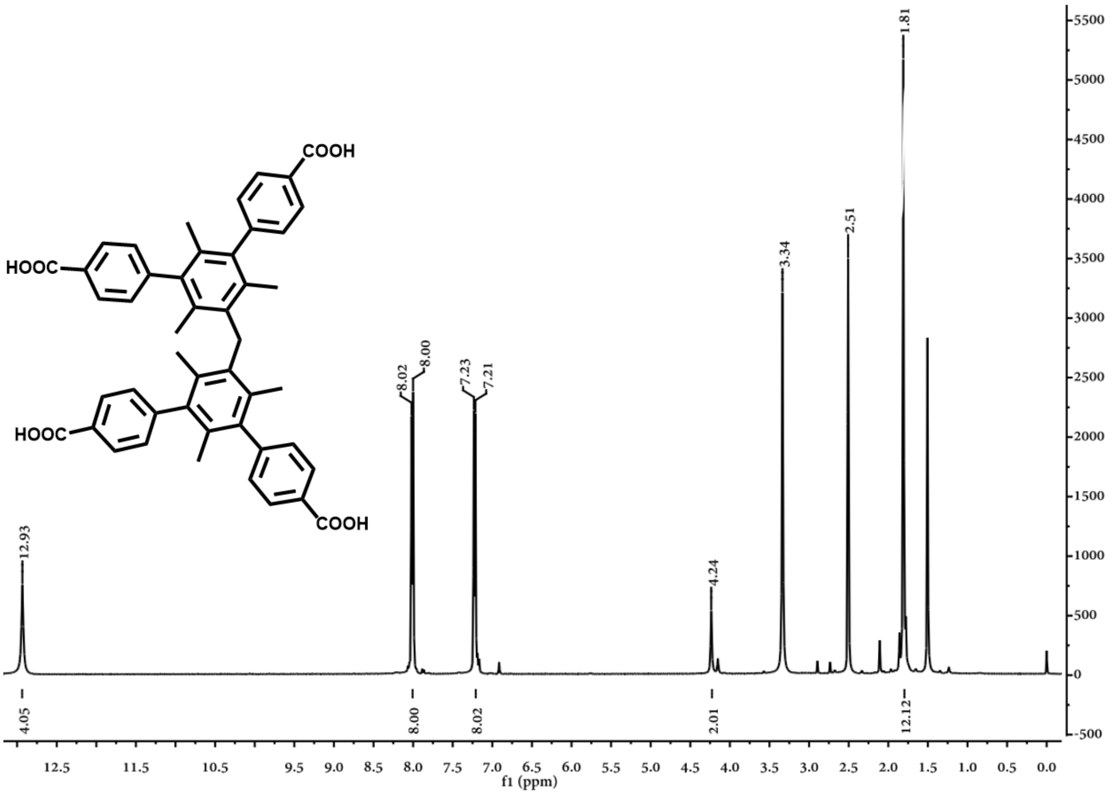

Figure S10. ^{1}H NMR of L-$(CH_3)_6$.

S2. MOF Synthesis

Synthesis of PCN-901(Zr)-SO_2. $ZrCl_4$ (20 mg), L-SO_2 (10 mg), and DMF (3 mL) were charged into a 10 mL vial, followed by the addition of 0.15 mL trifluoroacetic acid. The mixture was heated in 115 ºC oven for 2 days. After cooling down to room temperature, the colorless crystals of PCN-901(Zr)-SO_2 were harvested (yield: 75%).

Synthesis of PCN-902(Zr)-O. $ZrCl_4$ (20 mg), L-O (10 mg), and DMF (3 mL) were charged into a 10 mL vial, followed by the addition of 0.10 mL trifluoroacetic acid. The mixture was heated in 120 ºC oven for 2 days. After cooling down to room temperature, the colorless crystals of PCN-902(Zr)-O were harvested (yield: 70%).

Synthesis of PCN-902(Zr)-CH_2. $ZrCl_4$ (20 mg), L-CH_2 (10 mg), and DMF (3 mL) were charged into a 10 mL vial, followed by the addition of 0.10 mL trifluoroacetic

acid. The mixture was heated in 120 ºC oven for 2 days. After cooling down to room temperature, the colorless crystals of PCN-902(Zr)-CH_2 were harvested (yield: 71%).

Synthesis of PCN-903(Zr)-$(CH_3)_6$. $ZrCl_4$ (20 mg), L-$(CH_3)_6$ (10 mg), benzoic acid (500mg) and DMF (3 mL) were charged into a 10 mL vial. The mixture was heated in 120 ºC oven for 2 days. After cooling down to room temperature, the colorless crystals of PCN-903(Zr)-$(CH_3)_6$ were harvested (yield: 62%).

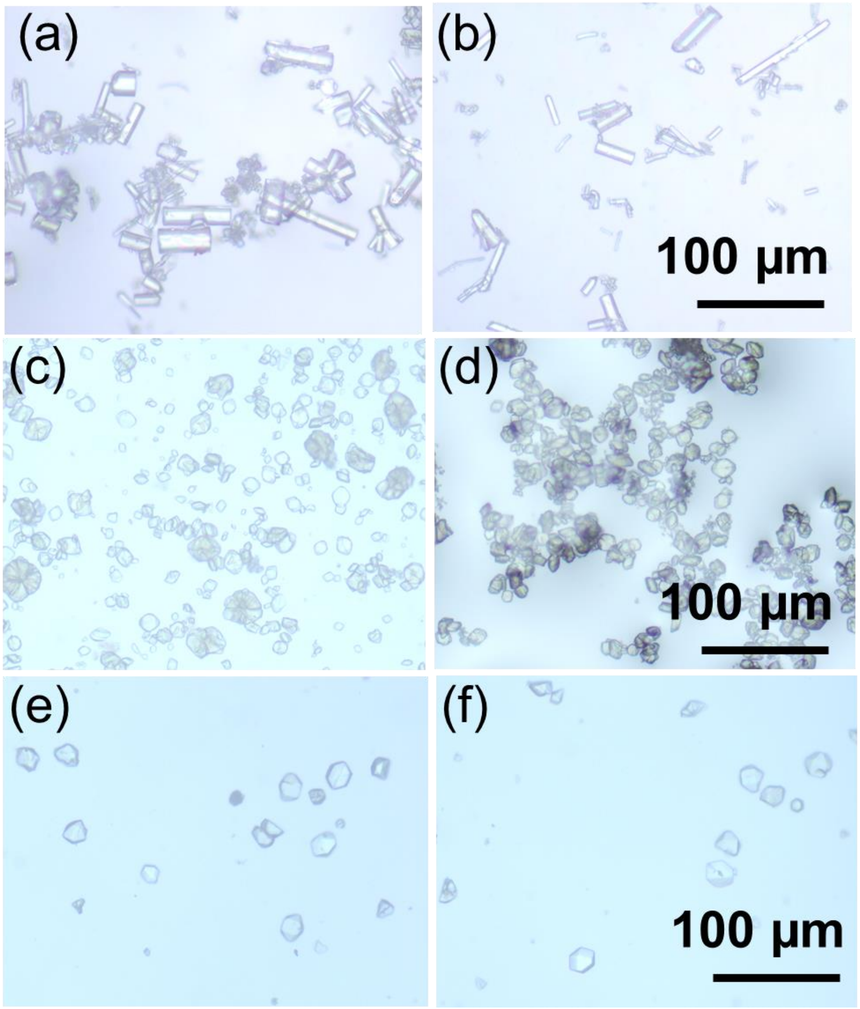

Figure S11. Optical images of (a-b) PCN-901(Zr)-SO_2, (c-d) PCN-902(Zr)-O and (e-f) PCN-903-$(CH_3)_6$ crystals.

S3. Single Crystal X-ray Crystallography

All as-synthesized crystals were taken from the mother liquid without further treatment, transferred to oil and mounted on to a loop for single crystal X-ray data collection. All crystals data were collected with a SuperNova diffractometer equipped with mirror Cu-Kα radiation (λ = 1.54184 Å) and an Eos CCD detector at 150 K. The data was collected with a ω-scan technique and an arbitrary φ-angle. Data reductions were performed with the CrysAlisPro package, and an analytical absorption correction was performed. All the structures were solved by the direct method using the *SHELXS* program of the *SHELXTL* package and refined by the full-matrix least-squares method with *SHELXL*.[2] The structures were treated anisotropically, whereas the aromatic and hydroxyl hydrogen atoms were placed in calculated ideal positions and refined as riding on their respective carbon or oxygen atoms. Structure was examined using the Addsym subroutine of PLATON to assure that no additional symmetry could be applied to the models. Crystal data collection are summarized in **Table S1**, Supporting Information and crystal structures can be accessed in CCDC 1937981-1937983.

Table S1. Crystal data and structure refinements.

Name	PCN-901-SO_2	PCN-902-O	PCN-903-$(CH_3)_6$
Empirical formula	$C_{37.75}H_{24}N_2O_{18}SZr_3$	$C_{40}H_{24}N_2O_{17}Zr_3$	$C_{33.5}H_{30}NO_{10.67}Zr_2$
Formula weight	1099.31	1096.29	800.19
Temperature/K	150	150	150
Crystal system	tetragonal	triclinic	trigonal
Space group	*P4/mmm*	*P-1*	*R-3c*
a/Å	28.491(4)	13.4104(7)	23.135(3)
b/Å	28.491(4)	18.7281(6)	23.135(3)
c/Å	15.714(4)	20.1519(5)	100.646(5)
α/°	90	78.966(2)	90
β/°	90	82.037(3)	90
γ/°	90	75.438(3)	120
Volume/$Å^3$	12756(5)	4786.2(3)	46653(13)
Z	4	2	36
ρ_{calc} g/cm^3	0.572	0.761	1.025
μ/mm^{-1}	2.355	0.356	0.441
F(000)	2178.0	1088	14538
Wavelength (Å)	1.54184	1.54184	1.54184
2Θ range for data collection/°	6.938 to 87.13	2.812 to 49.372	2.428 to 50.032
Reflections collected	4850	37752	35919
Independent reflections	2227	15666	8859
R_{int}	0.1419	0.1458	0.0441
Data	2227	15666	8859
Restraints	297	306	108
Parameters	151	559	458
GOF on F^2	0.967	1.655	1.050
$R1$ [I > 2σ(I)]	0.1146	0.1612	0.0850
wR2 [I > 2σ(I)]	0.3112	0.3894	0.2657
$R1$ (all data)	0.1818	0.2280	0.1088
wR2 (all data)	0.3823	0.4422	0.2928
Residue peak / hole (eÅ^{-3})	0.80/-0.77	4.65/-1.44	1.38/-0.64

S4. Computational Methods

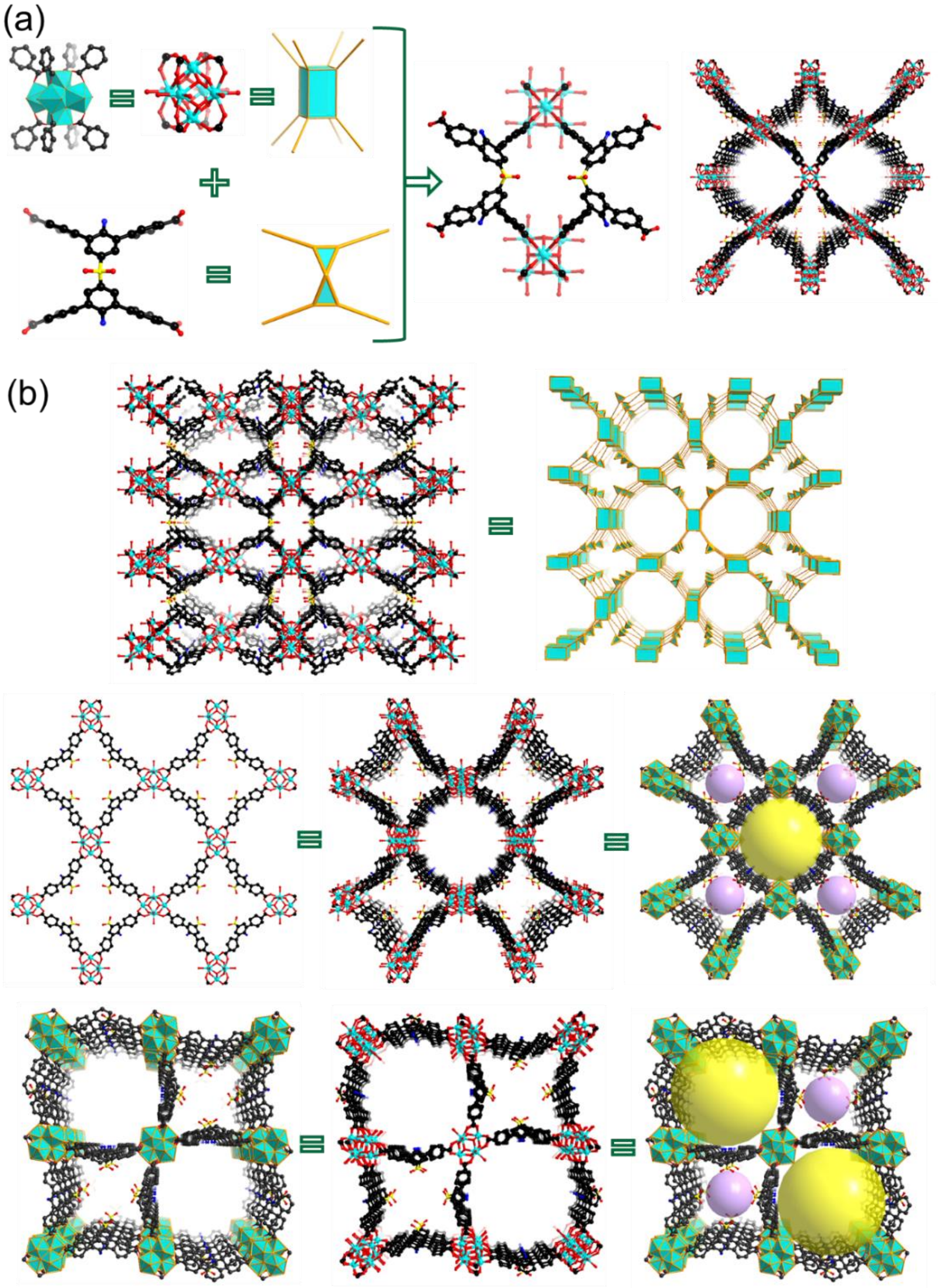

Figure S12. (a) Structural illustration of PCN-901-SO_2 with (4, 8)-c **scu** topology, constructed from 8-c Zr_6 clusters and 4-c linkers with C_{2v} symmetry; (b) Topology and porosity illustration of PCN-901-SO_2.

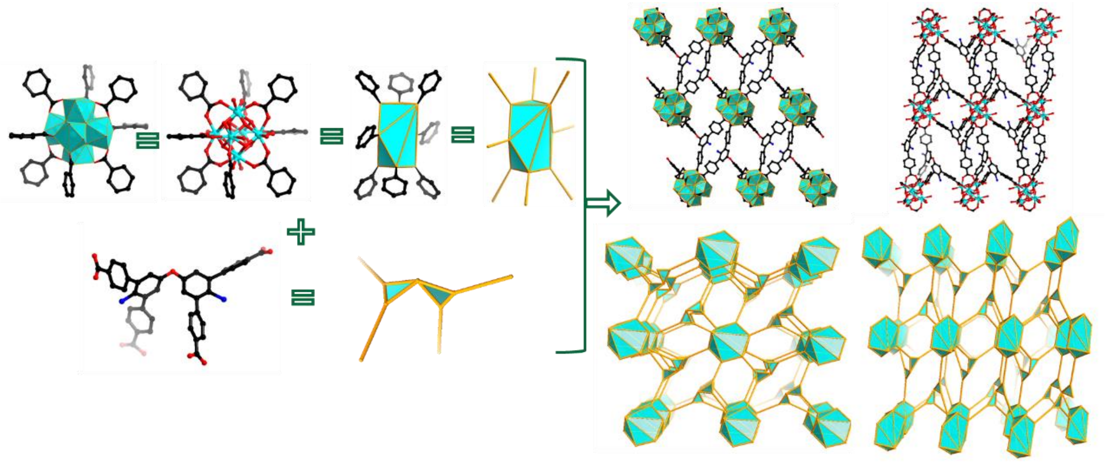

Figure S13. Structural and topological illustration of PCN-902-O with (4, 8)-c **flu** topology. The structure is constructed from unusual 8-c Zr_6 clusters and 4-c bent linkers.

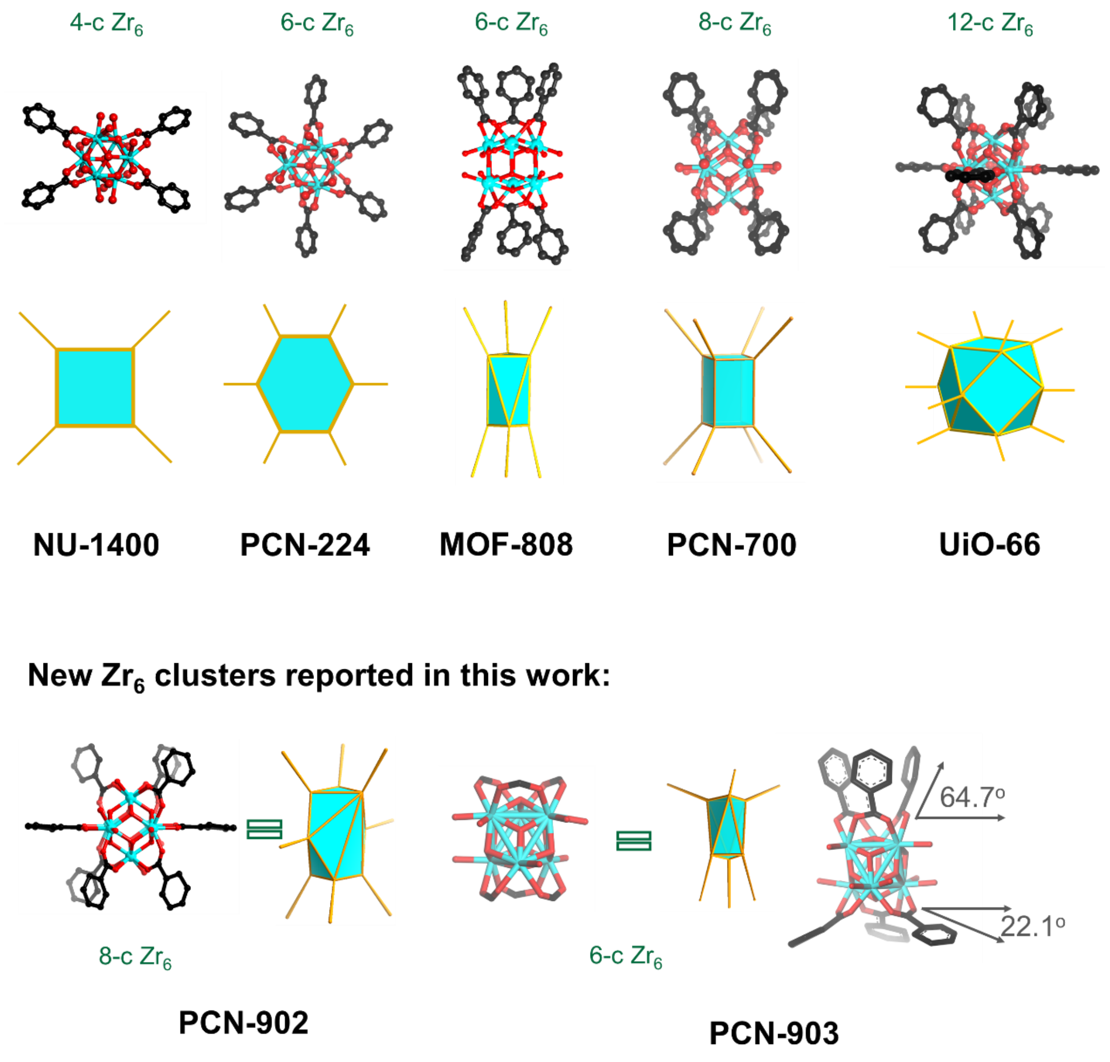

Figure S14. Cluster illustration of various Zr_6 clusters in literatures and in this work.

Computational Methods. To interpret the relationship between linker rigidness and the resulting topology, a density functional theory (DFT) study was performed by using DMol3 module of Material Studio program package.[3-4] The generalized gradient approximation (GGA) of the Perdew, Burke, Ernzerhof (PBE) functional and DNP 4.4 basis were employed for all calculations.[5] Grimme method was applied for DFT-D correction. To simulate the states of the linkers in PCN-901, -902 and -903, torsions between two lateral phenyl rings of ligands were constrained. For comparison, the energies of a given linker fragment in different conformations were normalized by subtracting the energy of its unconstrained structure. The calculated energies of linker fragments are listed in **Table S2**.

Table S2. Relative energies of ligands in different Zr-MOFs with unit of kJ/mol.

Ligand	Energy in PCN-901	Energy in PCN-902	Energy in PCN-903
L-SO_2	41.57	368.53	107.91
L-O	47.00	26.45	40.88
L-CH_2	34.45	22.64	37.81
L-$(CH_3)_6$	52.77	47.45	11.73

S5. Powder X-ray Diffraction

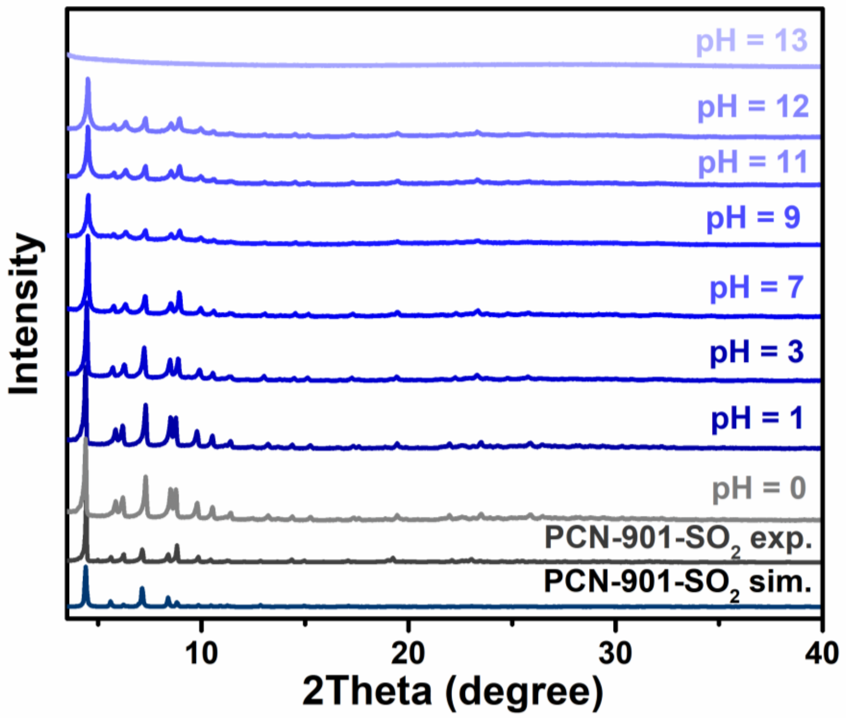

Figure S15. Powder X-ray diffraction (PXRD) patterns of PCN-901(Zr)-SO_2 after immersing in various aqueous solutions for 24 h.

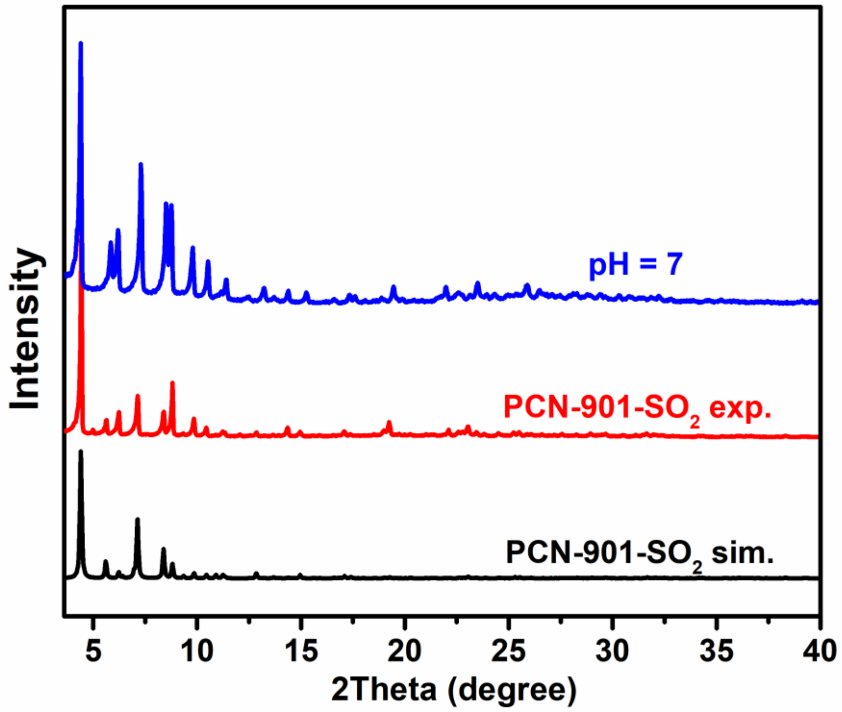

Figure S16. Powder X-ray diffraction (PXRD) patterns of PCN-901(Zr)-SO_2.

.

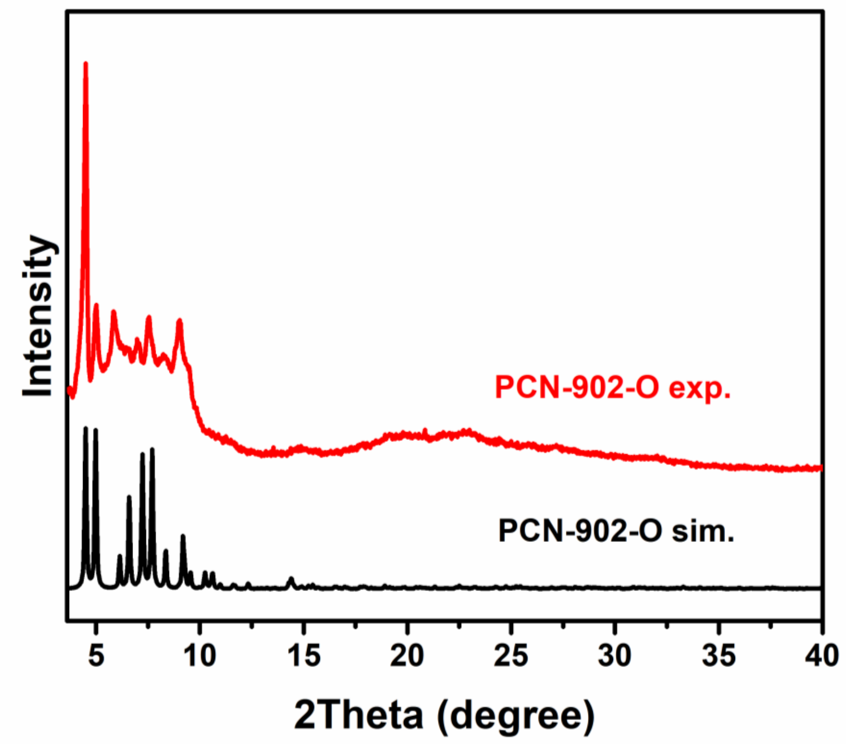

Figure S17. Powder X-ray diffraction (PXRD) patterns of PCN-902(Zr)-O.

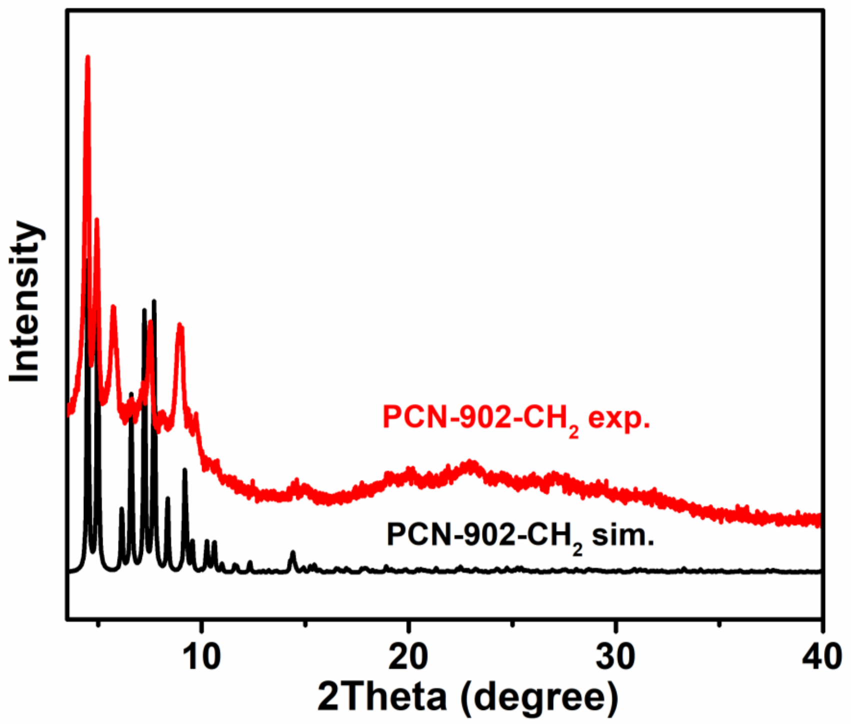

Figure S18. Powder X-ray diffraction (PXRD) patterns of PCN-902(Zr)-CH_2.

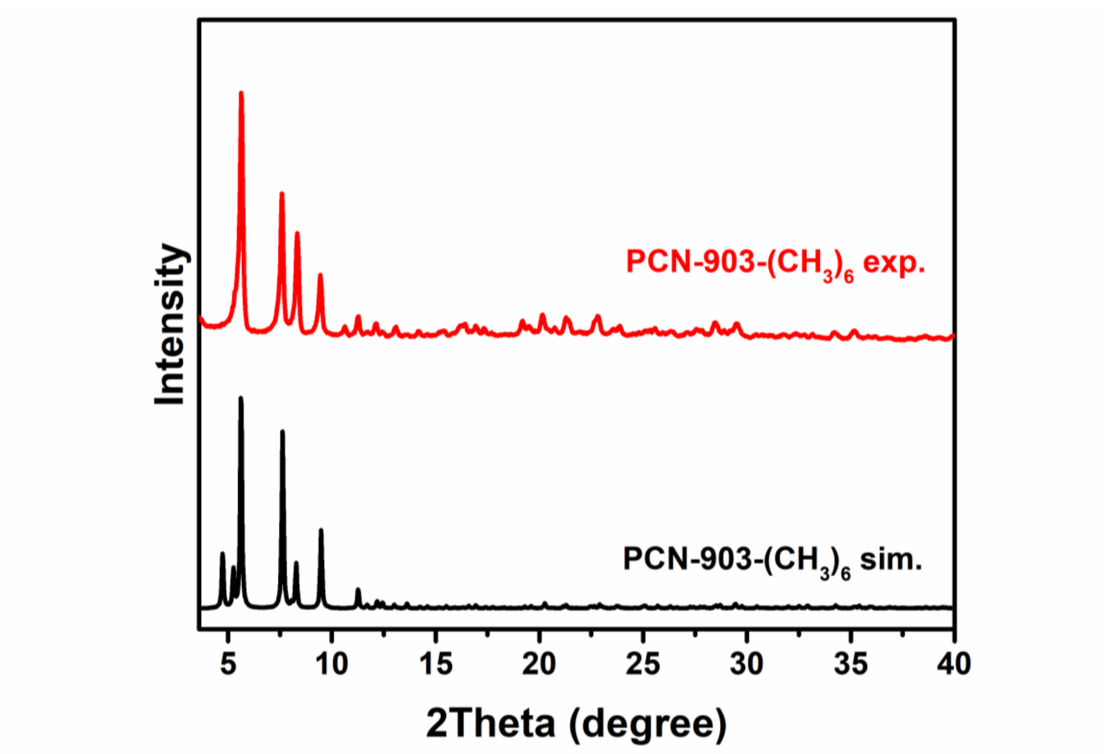

Figure S19. Powder X-ray diffraction (PXRD) patterns of PCN-903(Zr)-$(CH_3)_6$.

S6. Gas Sorption Isotherm

Before gas sorption experiment, as-synthesized sample was washed with DMF and immersed in CH_2Cl_2 and hexane for one day, during which the solvent was decanted and freshly replenished three times. The solvent was removed under vacuum at 100 ^{o}C, yielding a porous material. Gas sorption measurements were then conducted using a Micrometritics ASAP 2020 system.

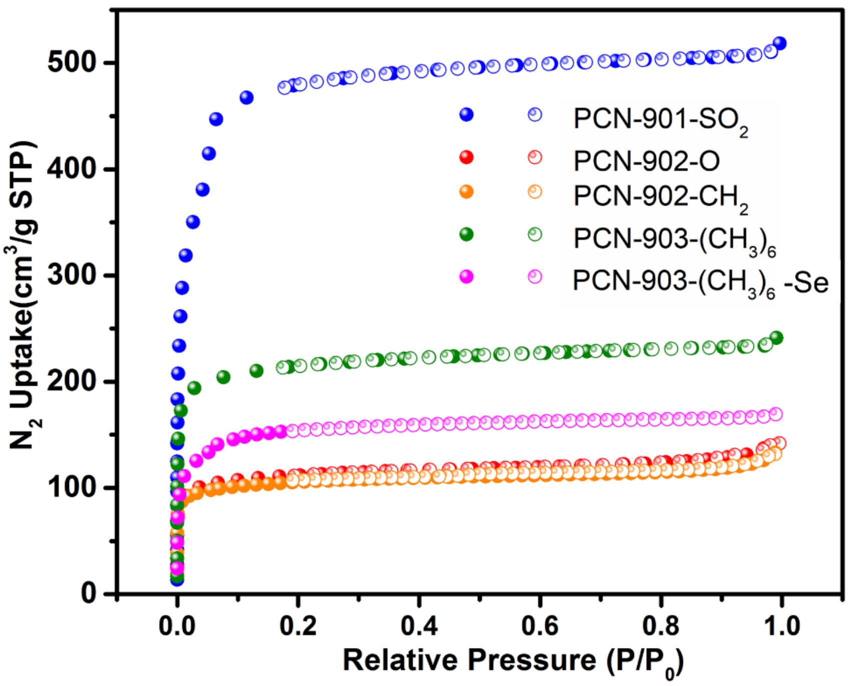

Figure S20. N_2 isotherms of PCN-901(Zr)-SO_2, PCN-902(Zr)-O, PCN-902(Zr)-CH_2, PCN-903(Zr)-$(CH_3)_6$ and PCN-903(Zr)-$(CH_3)_6$ after Se adsorption.

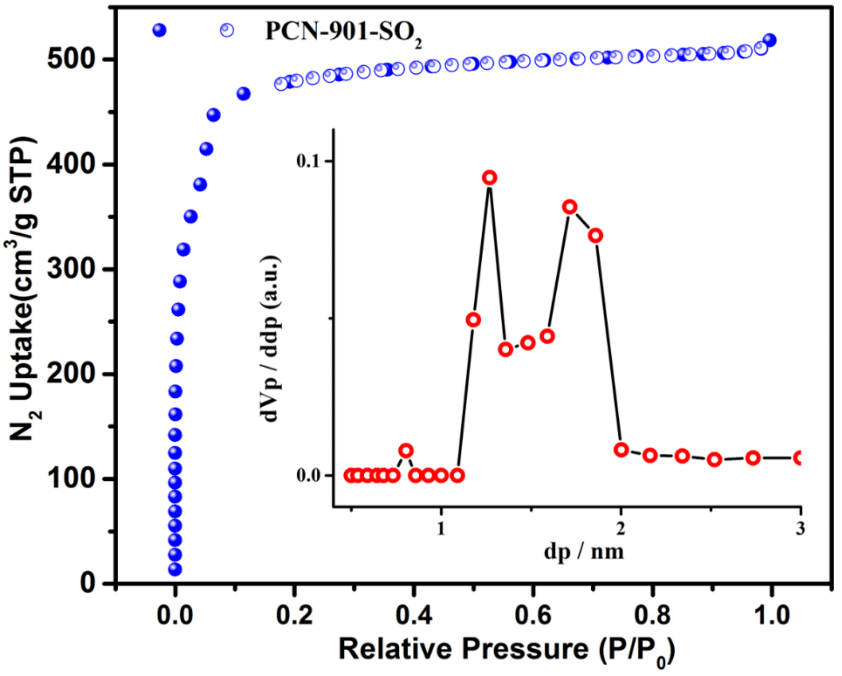

Figure S21. N_2 isotherm and the corresponding pore size distribution of PCN-901(Zr)-SO_2.

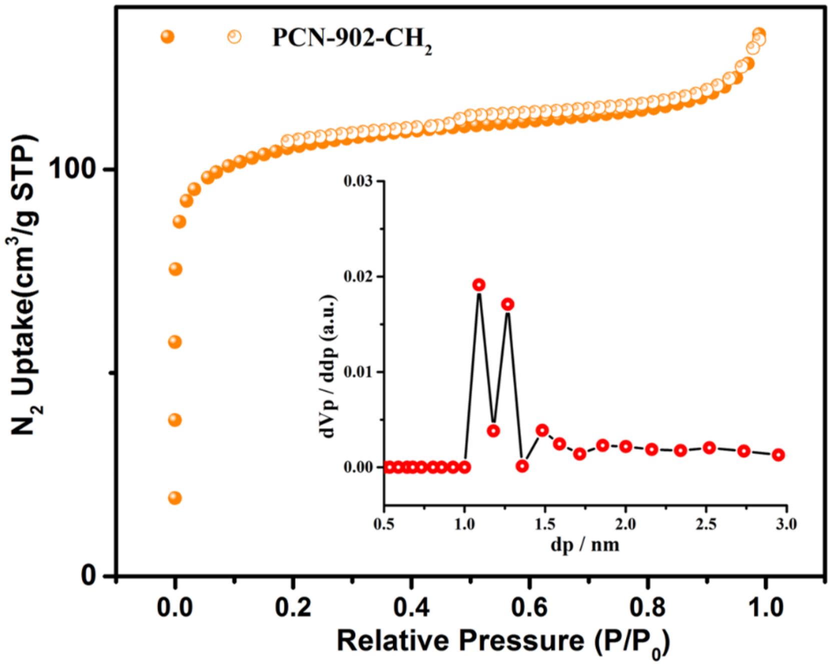

Figure S22. N_2 isotherm and the corresponding pore size distribution of PCN-902(Zr)-CH_2.

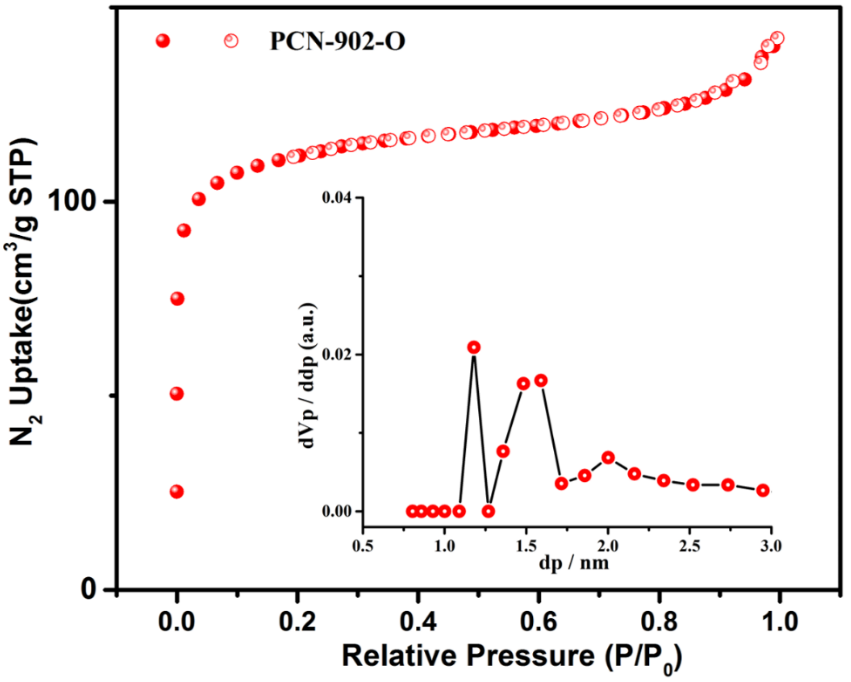

Figure S23. N_2 isotherm and the corresponding pore size distribution of PCN-902(Zr)-O.

Table S3. Porosity summary of PCN-901(Zr)-SO_2, PCN-902(Zr)-O, PCN-902(Zr)-CH_2, PCN-903(Zr)-$(CH_3)_6$ and PCN-903(Zr)-$(CH_3)_6$ after Se sorption.

MOF Sample	BET Surface Area (m^2/g)	Langmuir Surface Area (m^2/g)
PCN-901-SO_2	1782	2962
PCN-902-O	368	492
PCN-902-CH_2	340	466
PCN-903-$(CH_3)_6$	696	953
PCN-903-$(CH_3)_6$-Se	501	685

S7. Thermogravimetric Analysis

For thermogravimetric analysis, about 10 mg of the sample was heated on a TGA/DSC1 (Mettler-Toledo) thermogravimetric analyzer from 40 to 900 °C at a rate of 10 °C·min^{-1} under N_2 flow of 50 mL·min^{-1}.

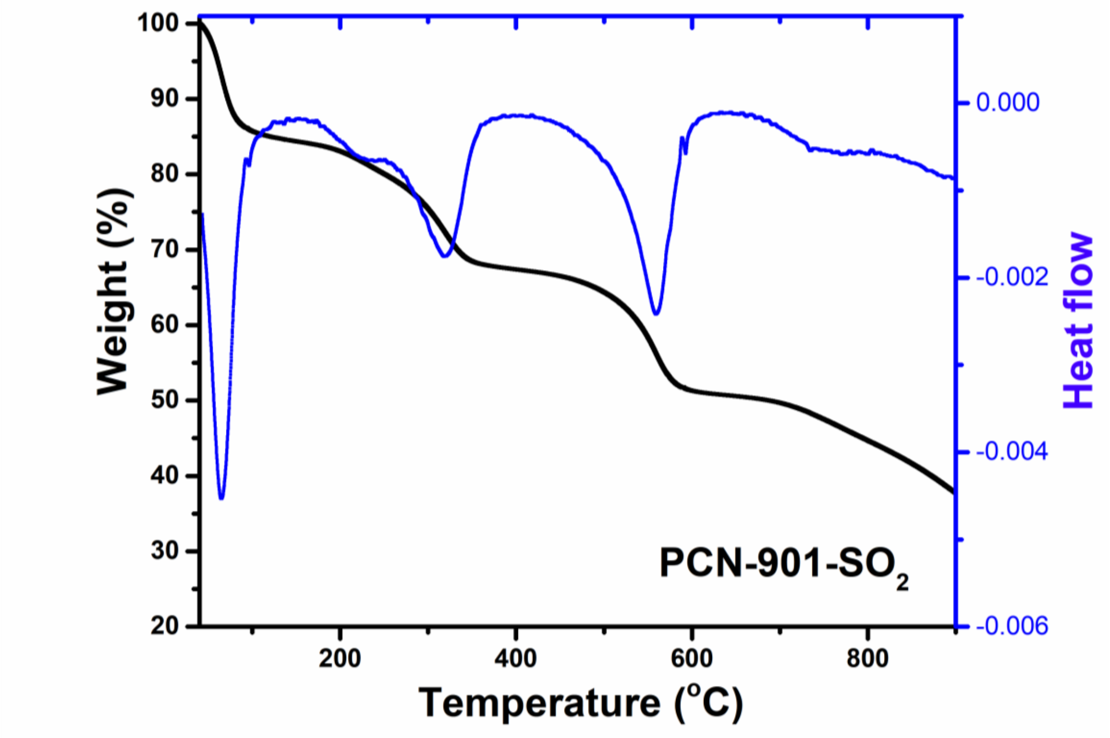

Figure S24. TGA-DSC plot of PCN-901-SO_2.

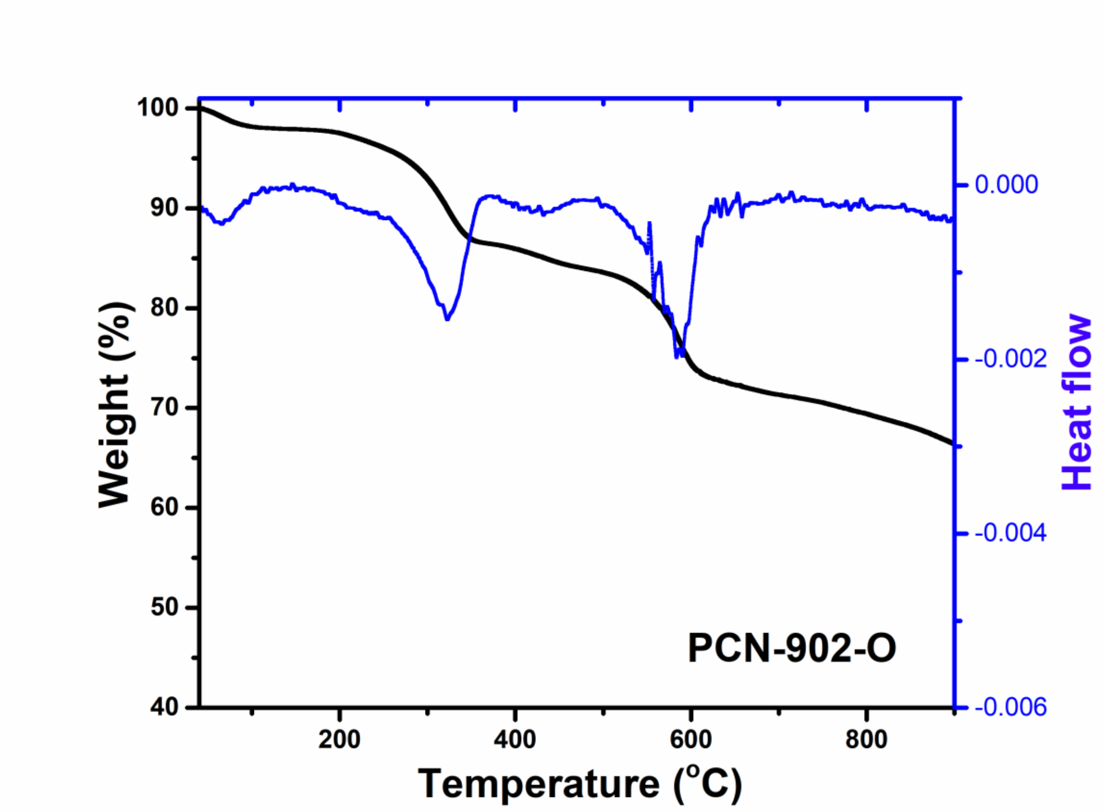

Figure S25. TGA-DSC plot of PCN-902-O.

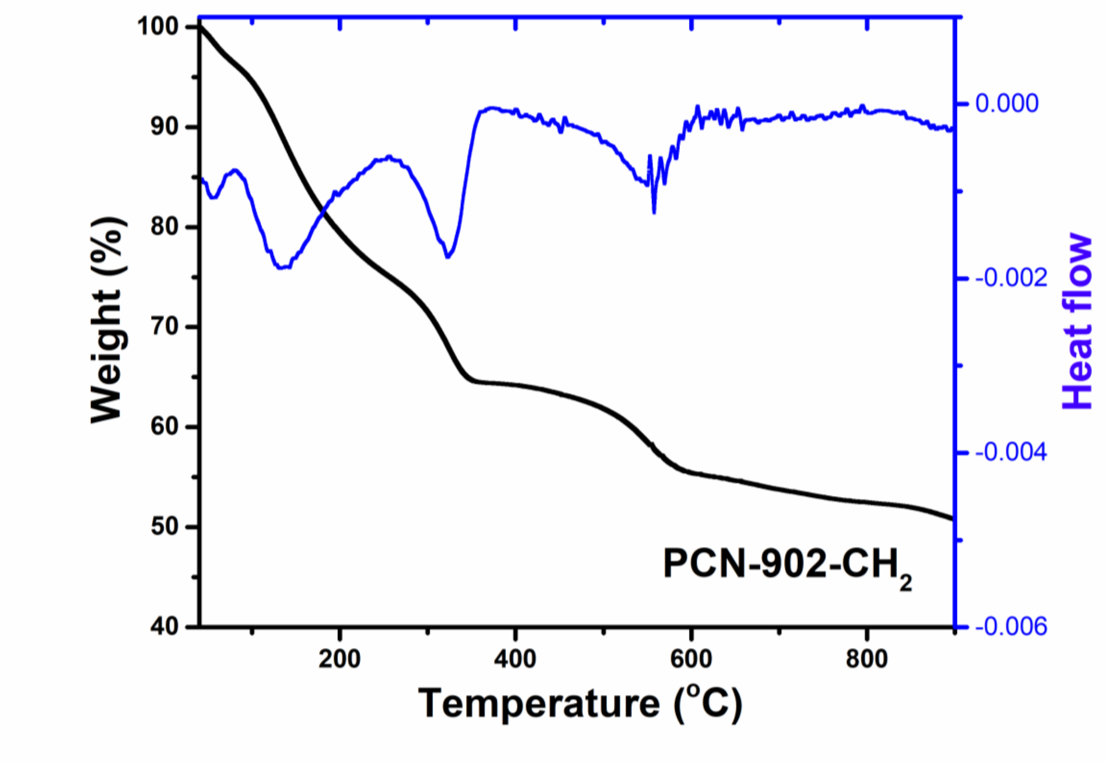

Figure S26. TGA-DSC plot of PCN-902-CH_2.

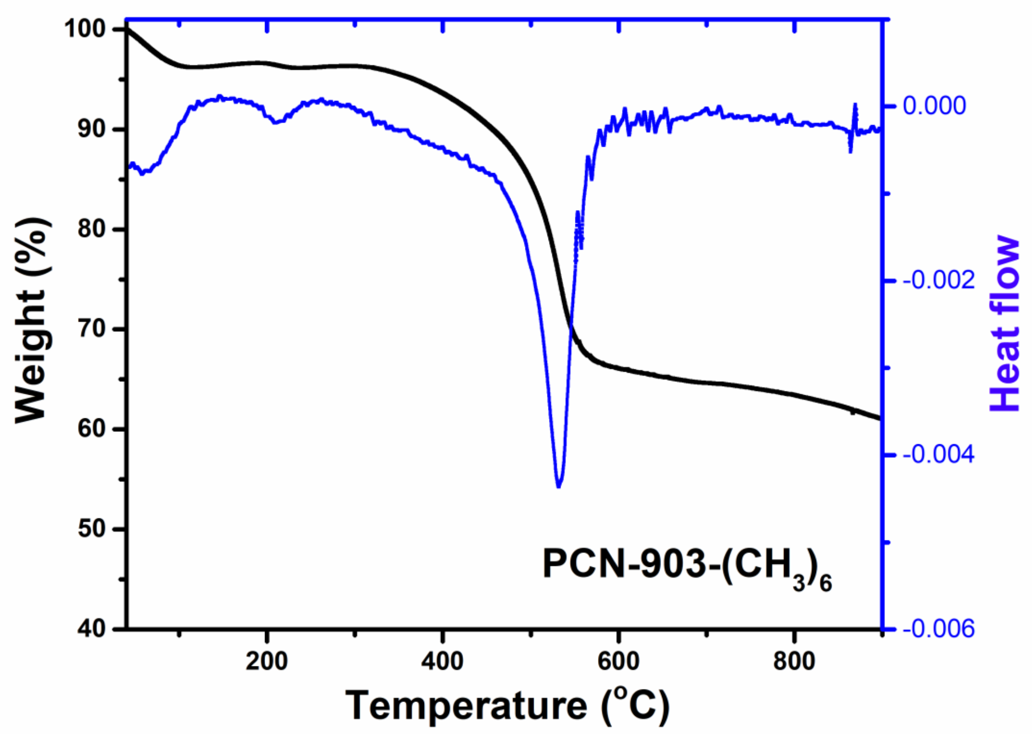

Figure S27. TGA-DSC plot of PCN-903-$(CH_3)_6$.

S8. Removal of Toxic Selenite Ions

Adsorption isotherm measurement. PCN-903-$(CH_3)_6$ (10 mg) was mixed with a 10 ml stock solution of selenite (SeO_3^{2-}) with various concentrations. The mixture was kept at room temperature for 24 h to ensure complete adsorption. The ion concentration in the supernatant solutions were determined by ICP-MS.

Adsorption kinetics measurement. PCN-903-$(CH_3)_6$ (10 mg) was mixed with a 10 ml stock solution of selenite (SeO_3^{2-}) at a concentration of 10 ppm. The mixture was kept at room temperature for various periods. During the adsorption process, the ion concentration in the supernatant solutions were analyzed by ICP-MS.

ICP-MS analysis. Calibration standards were prepared from certified reference standards from RICCA Chemical Company. Samples were further analyzed with a Perkin Elmer NexION® 300D ICP-MS. Additionally, in order to maintain accuracy, quality control samples from certified reference standards and internal standards were utilized.

Table S4 Sorption Data of PCN-903-$(CH_3)_6$ toward Se(IV) at Different Concentrations.[a]

c_0 (ppm)	c_f (ppm)	removal (%)	q_m (mg/g)
**10.0**	0.003	99.97	10.0
**20.0**	0.051	99.7	19.9
**50.0**	11.0	78.1	39.0
**100**	50.2	49.8	49.8
**200**	140	29.9	59.8
**500**	425	15.1	75.5

[a] m = 0.010 g, V = 10 mL, V:m = 1000 mL/g, 24 h contact time.

Table S5 Kinetic Data of Se(IV) Adsorption using PCN-903-$(CH_3)_6$.[a]

c_0 (ppm)	Time (min)	c_f (ppm)	removal (%)	K_d (mL/g)	q_m (mg/g)
**10.0**	7	0.981	90.2	9.17×10^3	9.0
	22	0.278	97.3	3.49×10^4	9.7
	40	0.125	98.7	7.84×10^4	9.8
	60	0.091	99.1	1.09×10^5	9.9
	120	0.019	99.8	5.26×10^5	10.0
	180	0.003	99.9	3.33×10^6	10.0

[a] m = 0.010 g, V = 10 mL, V:m = 1000 mL/g.

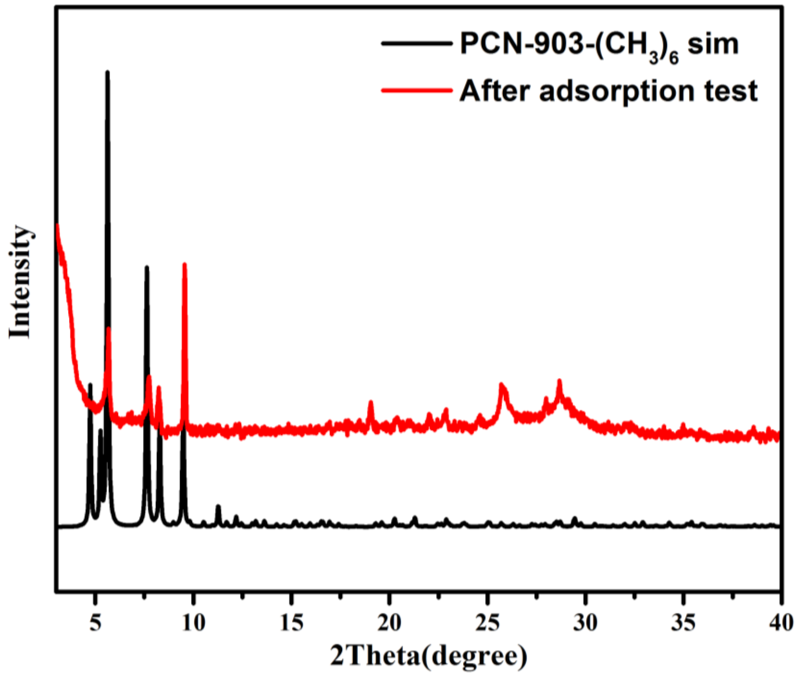

Figure S28. Powder X-ray diffraction (PXRD) patterns of PCN-903-$(CH_3)_6$ after Se adsorption test.

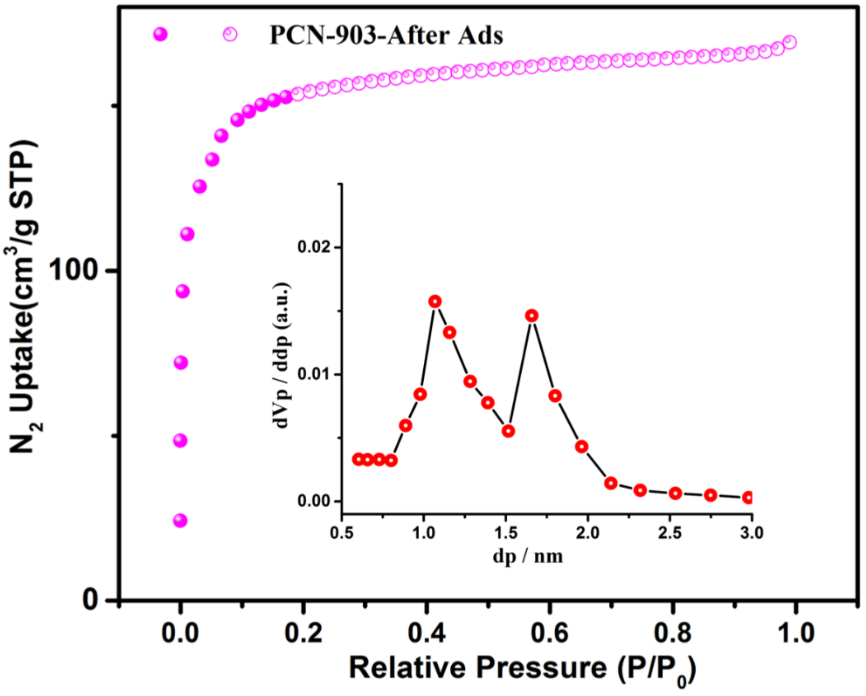

Figure S29. N_2 isotherm and the corresponding pore size distribution of PCN-903(Zr)-$(CH_3)_6$ after Se adsorption.

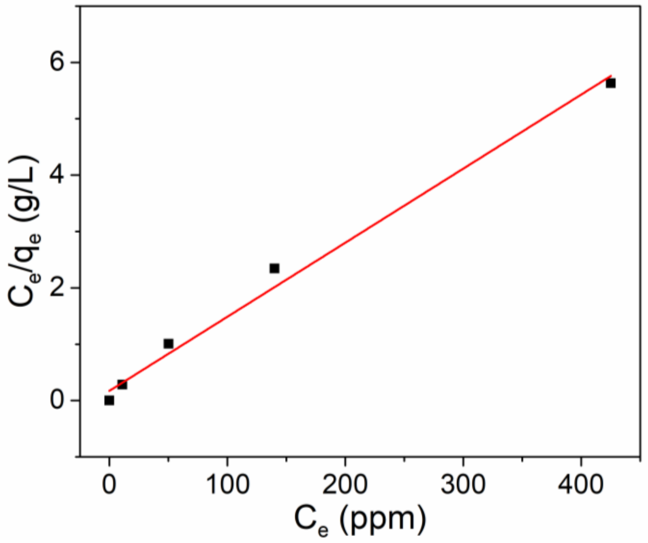

Figure S30. Langmuir plot for Se adsorption on PCN-903(Zr)-$(CH_3)_6$.

Table S6 Fitting results from Langmuir isotherm model.

Ion	Fitted q_m (mg/g)	K_L (L/mg)	R^2
Se(IV)	76.10	0.076	0.9888

Table S7 Maximum Adsorption Capacity of Various Adsorbents.

Name	Selenite Capacity (mg/g)	Time(min)	Ref
NU-1000	102	<1	6
CAU-17	150.0	<1	7
Mercapto-silica	7.9	<1	8
NanoFe	95.0	<1	9
Fe_3O_4@carbon microspheres	10.4	90	10
Aluminum Oxide on SiO_2	20	30	11
Iron Oxide on SiO_2	33	30	11
MIN	7.53	60	12
C-MIN	47.8	60	12
PCN-903	75.48	<1	This work

References

1. Omprakash G. Bhusnure, Sainath B. Zangade, Shivaji B. Chavan, Yeshwant B. Vibhute. *J. Chem. Pharm. Res.*, **2010**, 2(6), 234-243.
2. Daniel Teclechiel, Anna Christiansson, A Ke Bergman, Go Ran Marsh. *Environ. Sci. Technol*, **2007**, 41(21), 7459-7463.
3. Delley, B., *J. Chem. Phys.* **1990**, 92, 508-517;
4. Delley, B., *J. Chem. Phys.* **2000**, 113, 7756-7764.
5. Perdew, J. P.; Burke, K.; Ernzerhof, M., *Phys. Rev. Lett.* **1996**, 77, 3865-3868.
6. A. J. Howarth, M. J. Katz, T. C. Wang, A. E. P. Prats, K. W. Chapman, J. T. Hupp, O. K. Farha, *J. Am. Chem. Soc.* **2015**, 137, 7488-7494.
7. H. Ouyang, N. Chen, G. J. Chang, X. L. Zhao, Y. Y. Sun, S. Chen, *Angew. Chem. Int. Ed.* **2018**, 57, 13197-13201.
8. F. Sahin, M. Volkan, A. G. Howard, O. Ataman, *Talanta*, **2003**, 60, 1003-1009.
9. G. Zelmanov, R. Semiat, *Sep. Purif. Technol.* **2013**, 103, 167-172.
10. J. W. Lu, F. L. Fu, Z. C. Ding, N. Li, B. Tang, *J. Hazard. Mater.* **2017**, 330, 93-104.
11. Chan, Y. T.; Kuan, W. H.; Chen, T. Y.; Wang, M. K. *Water Res.* **2009**, 43, 4412-4420.
12. S. F. Evans, M. R. Ivancevic, J. Q. Yan, A. K. Naskar, A. M. Levine, R. J. Lee, C. Tsouris, M. P. Paranthaman. *Separation Science and Technology*, **2019**, 54, 2138–2146.
